# *Myrmozercon* mites are highly host specific: two new species of *Myrmozercon* Berlese associated with sympatric* Camponotus* ants in southern Quintana Roo, Mexico

**DOI:** 10.7717/peerj.18197

**Published:** 2024-10-25

**Authors:** Gabriela Pérez-Lachaud, Hans Klompen, Shahrooz Kazemi, Jean-Paul Lachaud

**Affiliations:** 1 Departamento Conservación de la Biodiversidad, El Colegio de la Frontera Sur, Chetumal, Quintana Roo, Mexico; 2 Department of Evolution, Ecology and Organismal Biology, Ohio State University, Columbus, OH, United States of America; 3 Department of Biodiversity, Institute of Science and High Technology and Environmental Sciences, Graduate University of Advanced Technology, Kerman, Iran

**Keywords:** Mesostigmata, Laelapidae, Melittiphinae, Formicidae, Camponotini, Neotropics, Myrmecophiles

## Abstract

Two new species of *Myrmozercon*, *M. serratus* sp. nov. and *M. spatulatus* sp. nov., are described based on adults and deutonymphs collected in association with ants in Mexico. They represent the first records of this genus from the Neotropic *s.s.* faunal region. Both new species are associated with hosts in the genus *Camponotus* from the same small area of a coastal lagoon, which share the same nesting habit preferences, providing strong evidence for host specificity. All but one colony of *C. atriceps* hosted mites, whereas they occurred in only half of the colonies of *C. rectangularis*. There was a significant positive correlation between the abundance of *C. atriceps* sexual ants and the abundance of mites. We summarize the known host associations for the genus *Myrmozercon* and discuss host specificity. Larvae of both mite species were collected on the wings of males and gynes suggesting that egg laying occurs on the hosts reproductive caste. Two hypotheses explaining this observation are discussed, larvae may be phoretic on winged sexuals, favoring mite co-dispersal with hosts, or larvae reside on the alates as a refuge from predation.

## INTRODUCTION

Ants (Hymenoptera: Formicidae) are key insect species that have multidimensional effects upon global biodiversity (Hölldobler & Wilson, 1990; Parker & Kronauer, 2021). Ants modify the environment while building complex nests with diverse microhabitats with relatively stable levels of temperature and humidity; the nests also contain plenty of resources. Ant colonies, their nests, and their surroundings, are therefore optimal microhabitats for a great number of other organisms, predominantly invertebrates (Hölldobler & Wilson, 1990; Hölldobler & Kwapich, 2022) and are considered as hot-points of diversity where species new to science can be discovered (Pérez-Lachaud & Lachaud, 2014). Mites are undoubtedly the most abundant and diverse symbionts (*s.l.*) in ant nests (Kistner, 1982; Rettenmeyer et al., 2011). About 20 families of the mite order Mesostigmata (superorder Parasitiformes) are known to be associated with ants (Hunter & Rosario, 1988; Walter & Proctor, 2013), with varying degrees of apparent specialization for ants and their nests (Hölldobler & Wilson, 1990; Gotwald Jr, 1997; Walter & Proctor, 2013).

Among Mesostigmata, the cosmopolitan Laelapidae is the most ecologically and morphologically diverse family, including free-living species, facultative and obligate parasites of a wide range of vertebrates, as well as arthropod symbionts (Evans & Till, 1965; Lindquist, Krantz & Walter, 2009; Kazemi, Rajaei & Beaulieu, 2014). With more than 1,300 described species in over 90 genera, the family is also one of the most speciose families of mesostigmatic mites (Beaulieu et al., 2011; Moraes et al., 2022). Moraes et al. (2022) considered 1,088 species assigned to 73 genera as free-living or arthropod associates with a few genera, such as *Holostaspis* Berlese (Babaeian, Mašán & Halliday, 2019), *Laelaspis* Berlese (Kazemi, 2015), and *Myrmozercon* Berlese (Hunter & Hunter, 1963; Joharchi, Babaeian & Seeman, 2015; Joharchi et al., 2023), apparently specialized for ant associations. The general biology and ecology, and specifically the feeding habits, of most ant associated laelapid species have rarely been studied, at least in part because of the challenges of studying very small animals inside the ant’s well defended fortresses. This is especially unfortunate, because those traits may vary significantly, even within genera (*e.g.*, Shaw, 2014). Second, with the exception of some economically important species, most of our knowledge of Laelapidae is restricted to descriptions of adults. Data on ecology, behavior and micro-habitat use of immatures is even more scarce than that for adults. The one generalization that appears to hold is that larvae in this family are short-lived and non-feeding, as laboratory studies on the feeding habits of species with potential as biological control agents such as *Cosmolaelaps vacua* (Michael) and *Hypoaspis larvicolus* Joharchi & Halliday have shown (Abou-Awad et al., 1989; Cakmak & Da Silva, 2018).

Among Laelapidae, the melittiphine genus *Myrmozercon* Berlese is one of the most morphologically diverse genera. It includes 28 described extant and one (undescribed) extinct (Dunlop et al., 2014) species (Moraes et al., 2022; Joharchi et al., 2023). Nearly all species have been collected with ants or are considered as associated with ants. A single species, *M. chapmani* (Baker & Strandtmann) was described from two females discovered on orchid plants from Mexico City intercepted at the U.S.-Mexico border (Baker & Strandtmann, 1948; Hunter & Hunter, 1963). This species is currently the only one known from Mexico, but its precise origin remains unclear. Herein, we describe two new species of *Myrmozercon* associated with two sympatric formicine ant species of the genus *Camponotus* Mayr in the state of Quintana Roo, in southern Mexico. To our knowledge, these are the first reliable records of this genus from the Neotropic *s.s.* faunal region. In addition, we discuss host specificity and *Myrmozercon* diversity, as well as possible explanations for the unexpected presence of mite larvae on the wings of ant alates.

## MATERIALS & METHODS

### Study site

**Figure 1:**
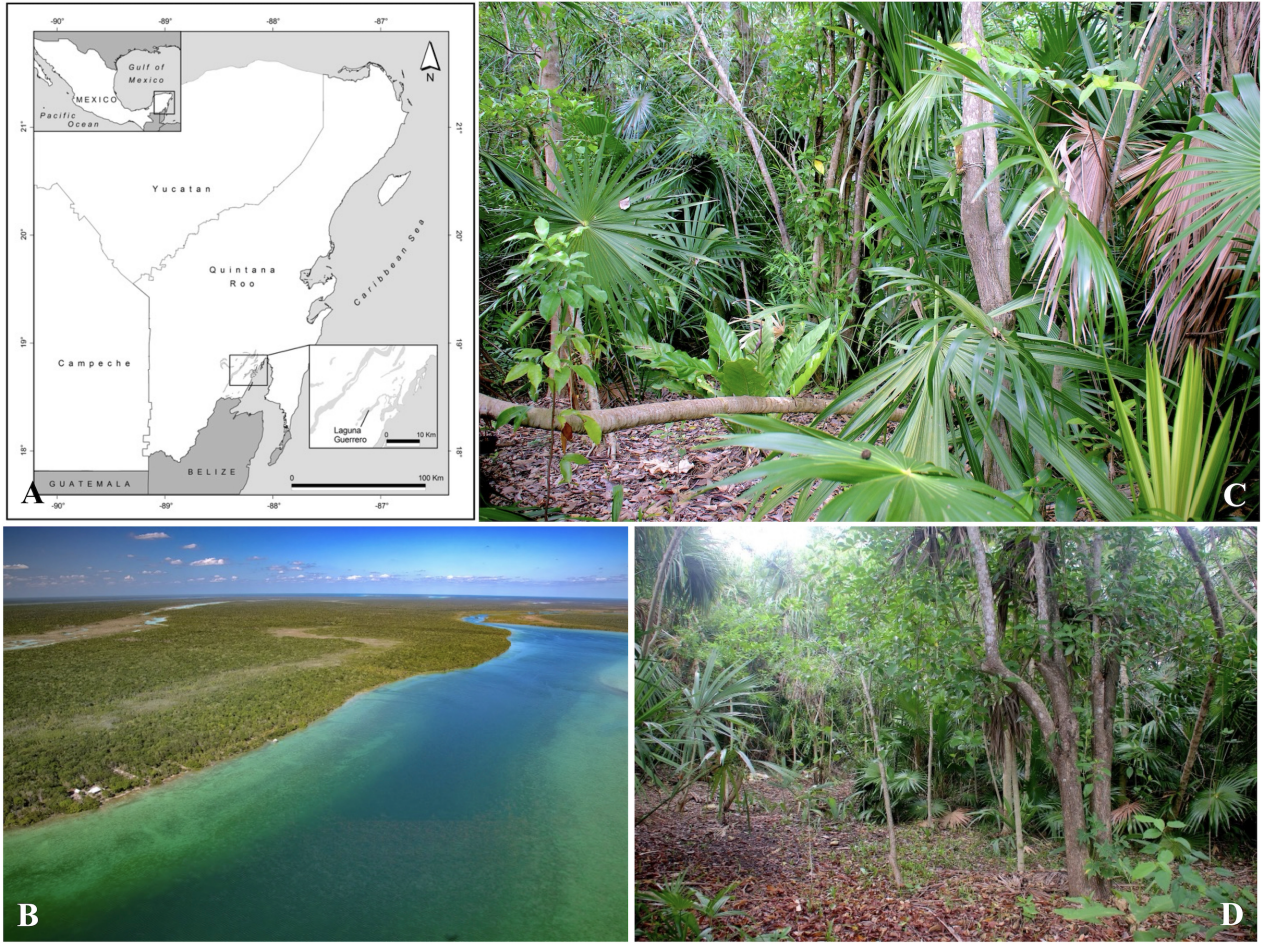
Study site (Laguna Guerrero). (A) Map of the study site (credit: Holger Weissenberger). (B) General view of the mangrove and low vegetation at the border of the lagoon. (C–D) Views of vegetation at the private site. Photos credit: Humberto Bahena-Basave (B); Jean-Paul Lachaud (C–D).

Ants and mites were collected in a 2,000 m^2^ coastal lagoon private site, located at Laguna Guerrero (18°41′31.2″N, 88°15′41.4″W), 21 km NE of Chetumal, in the southern portion of Quintana Roo, Mexico (Fig. 1). Mangrove trees border the lagoon, and indigenous trees and palms are intermixed with coconut palm trees and ornamental plants (for more details on the vegetation composition, see Supplemental Information 1, Figs. S1–S2, and the study site description in Pérez-Lachaud & Lachaud, 2021). The climate of the region is of the Aw type, warm sub-humid, with rainfall during the summer and the driest period during March and April, according to the classification of Köppen as modified by García (1973).

### Sampling

Several adult mites were initially observed within a colony of the arboreal formicine ant, *Camponotus rectangularis* Emery, which occupied a bamboo trap-nest set up as part of a larger project focused on parasitoids and other myrmecophiles associated with ants (see Pérez-Lachaud & Lachaud, 2021). Interestingly, a mite larva was found attached to the wings of a gyne (alate female) suggesting phoretic behavior; this prompted our attention to mites. Six colonies (or parts of colonies) were collected in 2020 as part of the aforementioned study. To verify the specificity and nature of the mite/ant association we collected three additional colonies of *C. rectangularis* and several colonies of another *Camponotus* species, *C. atriceps* (F. Smith), at the same site, between August 2020 and February 2024. *Camponotus rectangularis* is an arboreal, opportunistic cavity breeder (Wheeler, 1934; Durou et al., 2002), frequently associated with epiphytes (Dejean, Olmsted & Snelling, 1995), and with seemingly polydomous nesting habits (Pérez-Lachaud & Lachaud, 2021); *C. atriceps* is very abundant in the study site, nesting almost anywhere in second growth vegetation, in dead wood on the ground, or in live branches of *Cecropia*, and has a polydomous colony structure at least in southern Mexico (Longino, 2002; J-P Lachaud & G Pérez-Lachaud, pers. obs., 2021). The two species commonly use preformed cavities in dead wood as nesting sites. Complete colonies or samples were collected using artificial, bamboo made, trap-nests as in Pérez-Lachaud & Lachaud (2021) or by actively searching ants in dead branches and dried pseudobulbs of *Myrmecophila tibicinis* (Boneman ex Lindley) Rolfe (Orchidaceae).

Collected material (ants and nesting supports) was kept in a fridge before inspection. Dry branches were cut open, and ants and their brood were collected with forceps. The content of trap nests and orchid pseudobulbs was directly transferred to a jar with 96° alcohol. Preserved material was sorted and counted under a stereomicroscope. Additionally, preserved material stored in our collection (belonging to several other arboreal ant species, collected at the same site) was examined in search for associated mites (see Results, Table S1).

Mites were examined using a Nikon SMZ-745T dissecting stereomicroscope (6.3–100X), and a JEOL-JSM6010 scanning electron microscope (SEM). For SEM analysis, specimens were dehydrated in a graded ethanol series from 70 to 100%, left to dry at room temperature, fixed on stubs, and sputter coated with gold before observation. Specimens were initially identified as belonging to the Laelapidae with available keys; representative material was sent to HK and SK who confirmed the specimens as belonging to *Myrmozercon*. Images were taken at OSAL using the automated Z-stacking feature of the Nikon NIS Elements package on a Nikon Eclipse 90i (Melville, NY, USA) compound microscope with a PC controlled Ds-5M-U1 digital camera. The morphological terminology for the mites mostly follows Evans & Till (1965) for the body, Evans (1963a), Evans (1963b) for the leg and palp chaetotaxy, and Kazemi (2020) for supralabral process. All measurements are in micrometers (µm).

Five specimens were used for DNA extraction using a standard glass fiber method (Ivanova, Dewaard & Hebert, 2006). Polymerase chain reactions were performed to amplify the mitochondrial cytochrome c oxidase subunit I (COI) gene. PCR protocols follow Montes-Ortiz & Elías-Gutiérrez (2018). PCR products were sent for sequencing to Eurofins Genomics, LLC, Louisville, KY, USA. Sequences were edited using CodonCode v. 3.0.1 (CodonCode Corporation, Dedham, MA, USA) and uploaded to the Barcode of Life Database (BOLD, boldsystems.org, dataset DS-MITEQROO) and to GenBank (accession numbers PP941117 and PP941118). Specimens were recovered after the lysis step from the glass fiber filter plate, preserved in 96% ethanol, and deposited as vouchers. Representative specimens of ants and mites were deposited in the Formicidae and Arthropoda collections of El Colegio de la Frontera Sur at Chetumal, Quintana Roo, Mexico (ECO-CH-F and ECO-CH-AR, respectively), in the Colección Nacional de Ácaros, Instituto de Biología, Universidad Nacional Autónoma de México, Mexico City, Mexico (CNAC), and in the Ohio State University Acarology Collection (OSAL). Field sampling complied with the current laws of Mexico and was carried out under permit number FAUT-0277 issued to GP-L by the Secretaría de Medio Ambiente y Recursos Naturales, Dirección General de Vida Silvestre, Mexico.

The electronic version of this article in Portable Document Format (PDF) will represent a published work according to the International Commission on Zoological Nomenclature (ICZN), and hence the new names contained in the electronic version are effectively published under that Code from the electronic edition alone. This published work and the nomenclatural acts it contains have been registered in ZooBank, the online registration system for the ICZN. The ZooBank LSIDs (Life Science Identifiers) can be resolved and the associated information viewed through any standard web browser by appending the LSID to the prefix http://zoobank.org/. The LSID for this publication is: urn:lsid:zoobank.org:pub:93B2E7CB-3C56-4C21-922A-B4B6FCABB3DF. The online version of this work is archived and available from the following digital repositories: PeerJ, PubMed Central SCIE and CLOCKSS.

**Figure 2:**
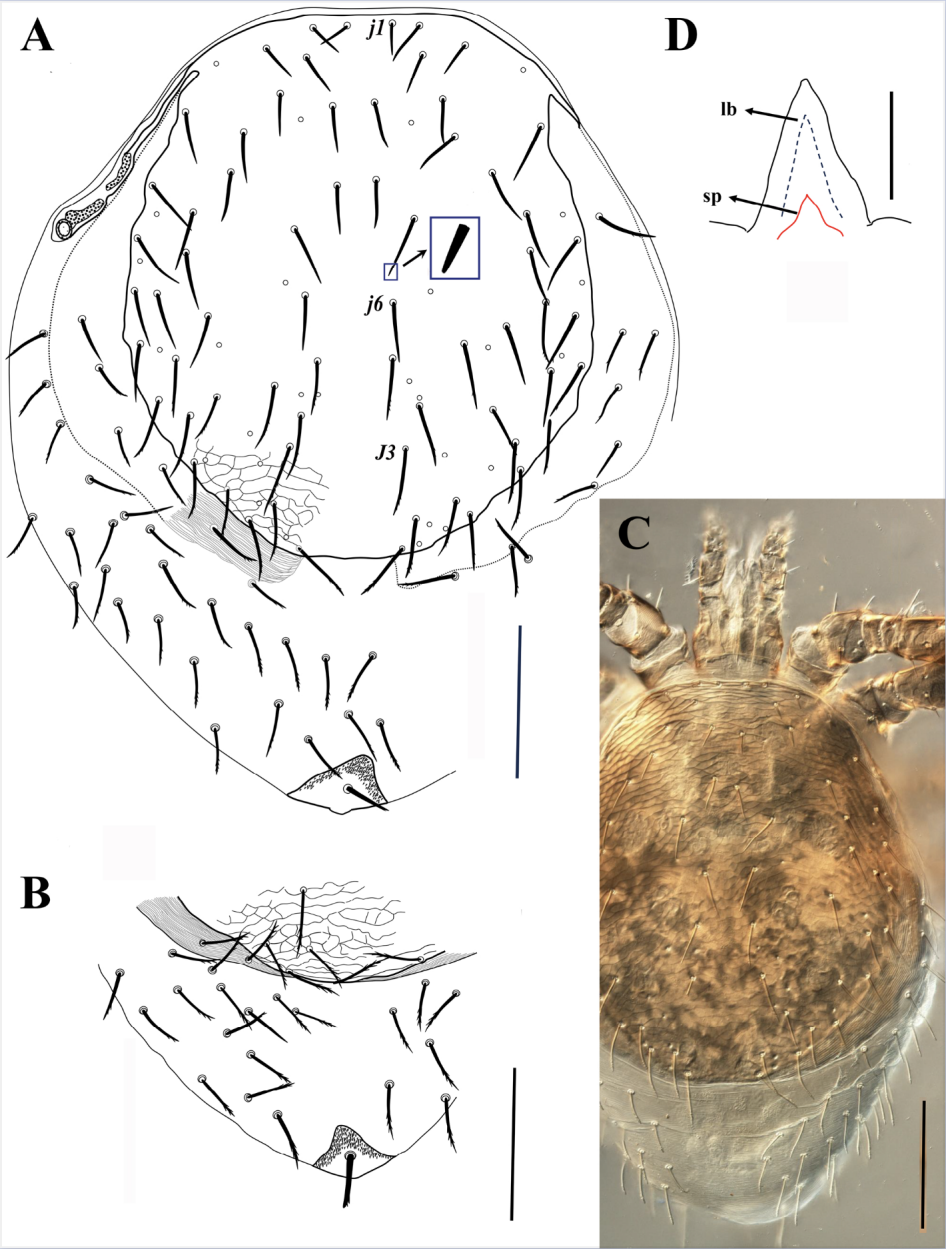
*Myrmozercon serratus* sp. nov., female. (A, C) Dorsal views. (B) Alternative arrangement of sclerotized band of marginal striations. Scale bar: 200 µm. (D) Gnathotectum. Scale bar: 50 µm; abbreviations: *lb*, labrum; *sp*, supralabral process. Photo credit: Shahrooz Kazemi & Hans Klompen.

**Figure 3:**
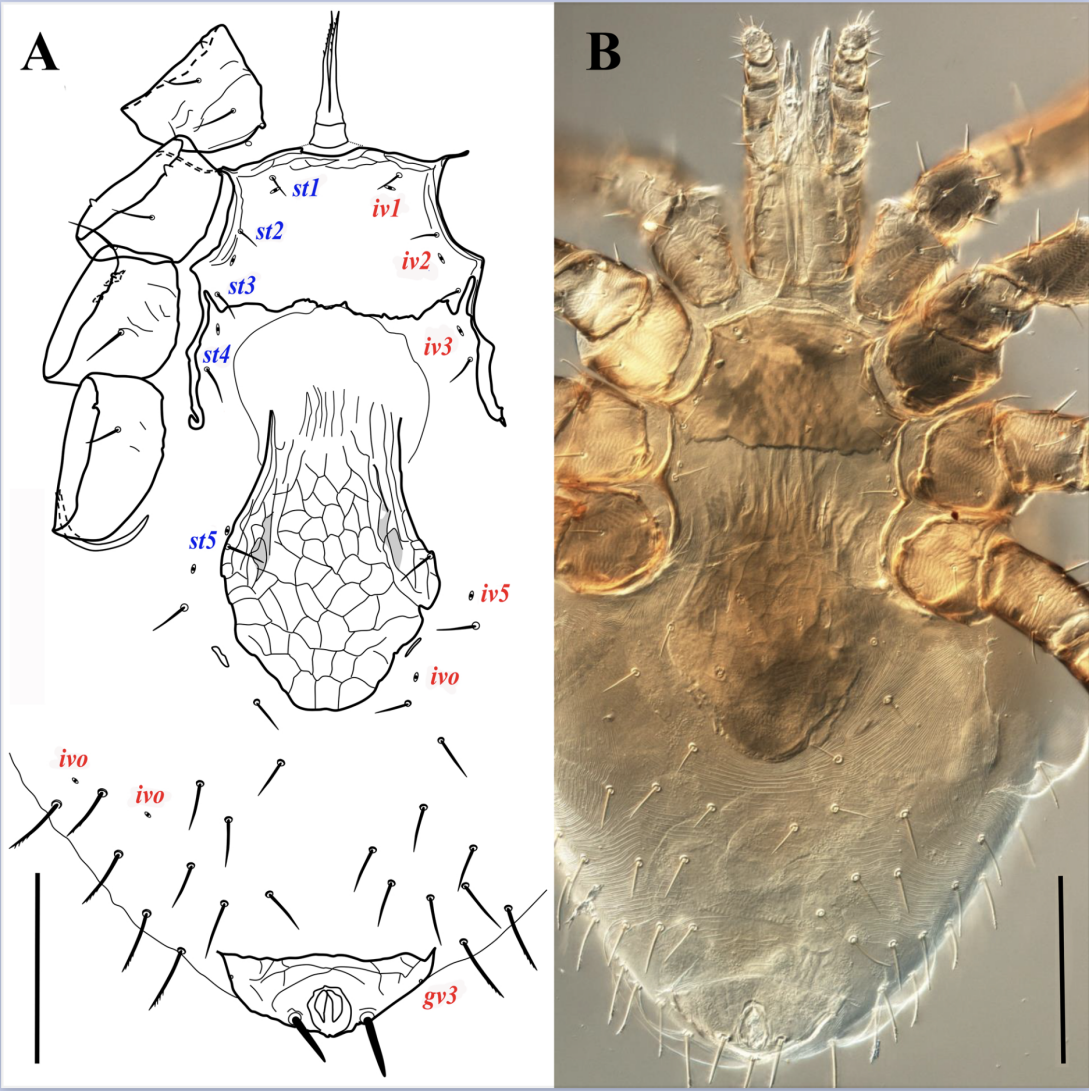
*Myrmozercon serratus* sp. nov., female. (A, B) Ventral views. Scale bar: 200 µm. Photo credit: Shahrooz Kazemi & Hans Klompen.

**Figure 4:**
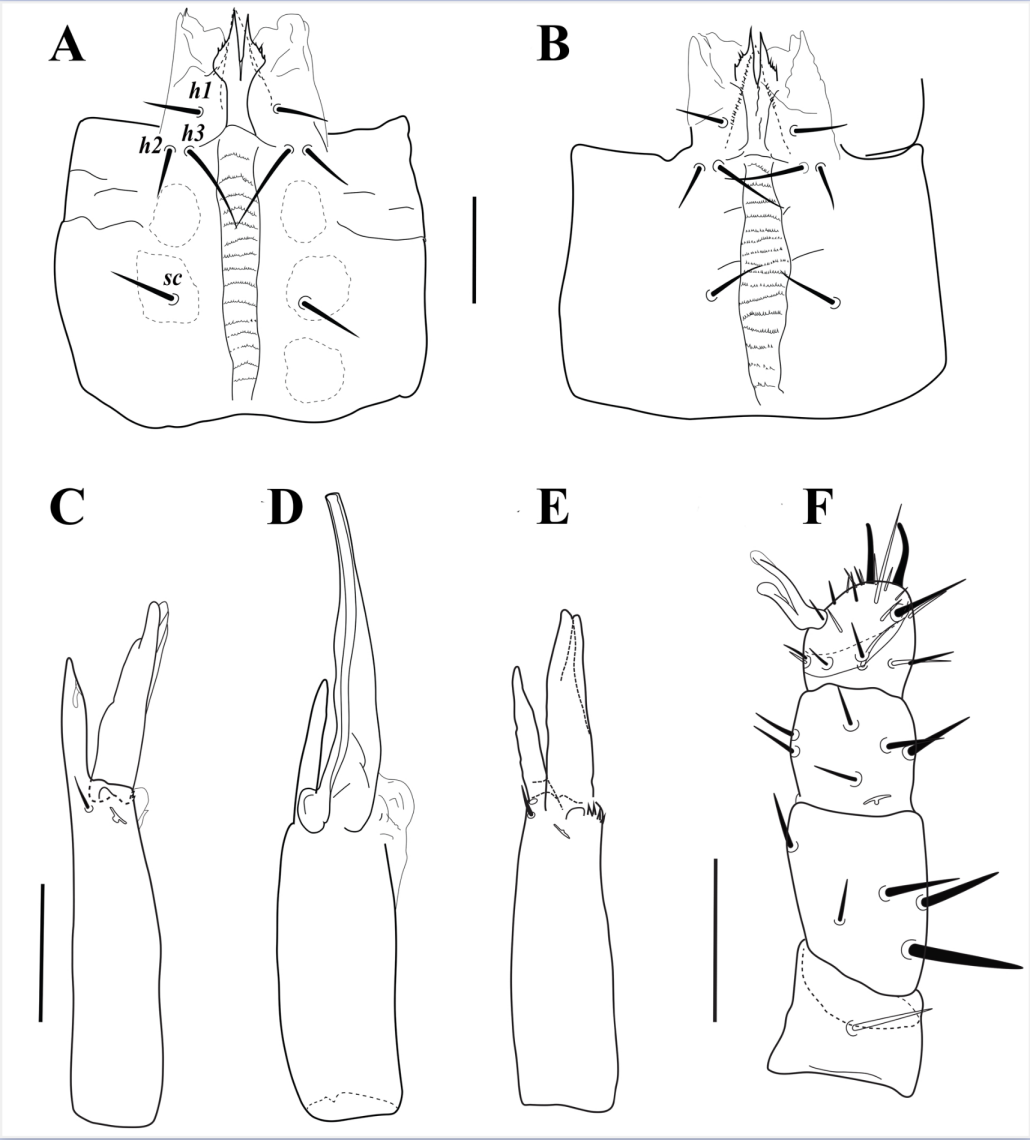
*Myrmozercon serratus* sp. nov. (A) Subcapitulum female. (B) Subcapitulum deutonymph. (C) Chelicera female. (D) Chelicera male. (E) Chelicera deutonymph. (F) Palp female. Scale bars: 50 µm.

**Figure 5:**
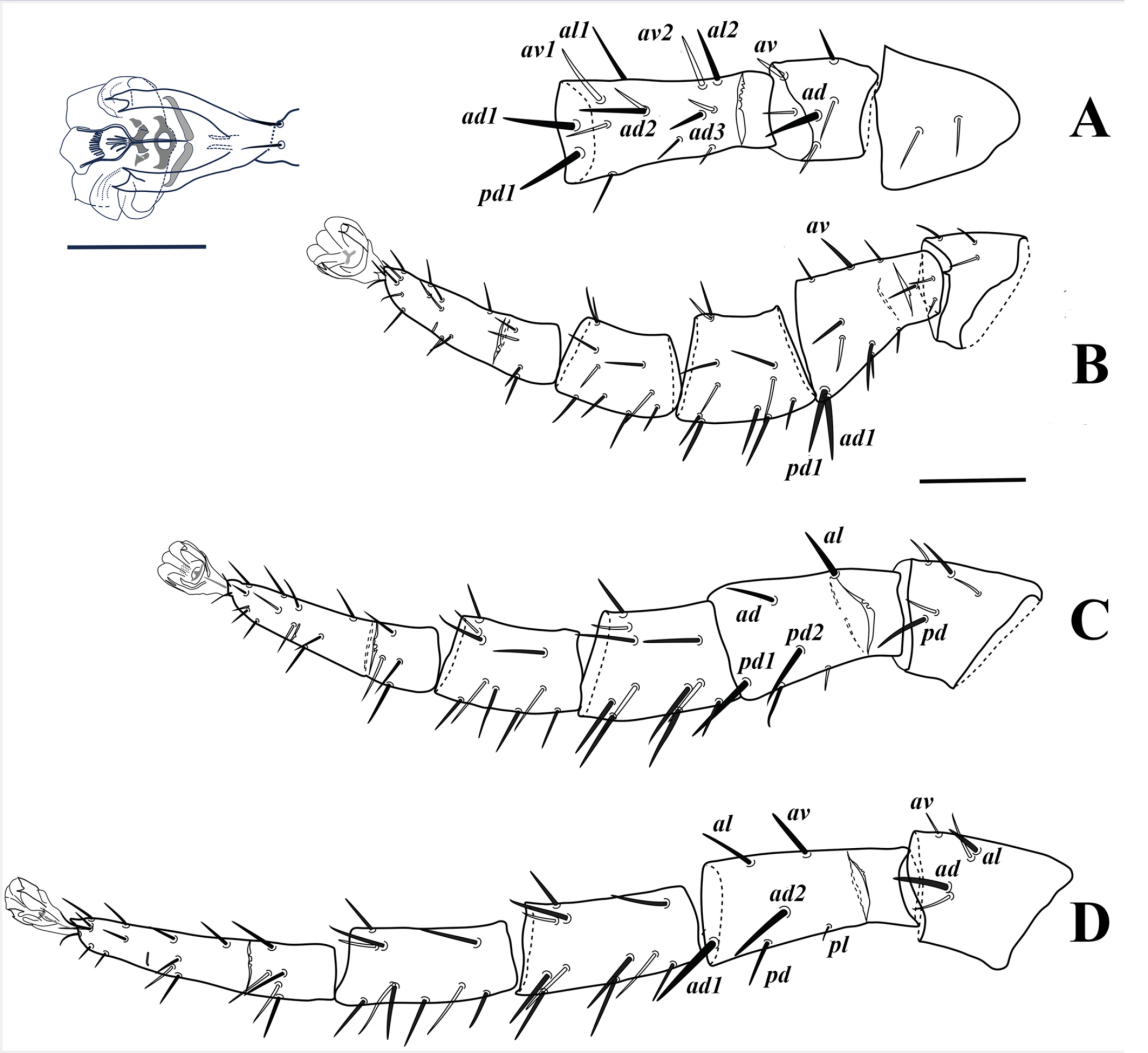
*Myrmozercon serratus* sp. nov., legs female, anterolateral view. (A) Partial leg I. (B–D) Leg II–IV. Scale bar: 100 µm. Inset, detail pretarsus IV; scale bar: 50 µm.

**Figure 6:**
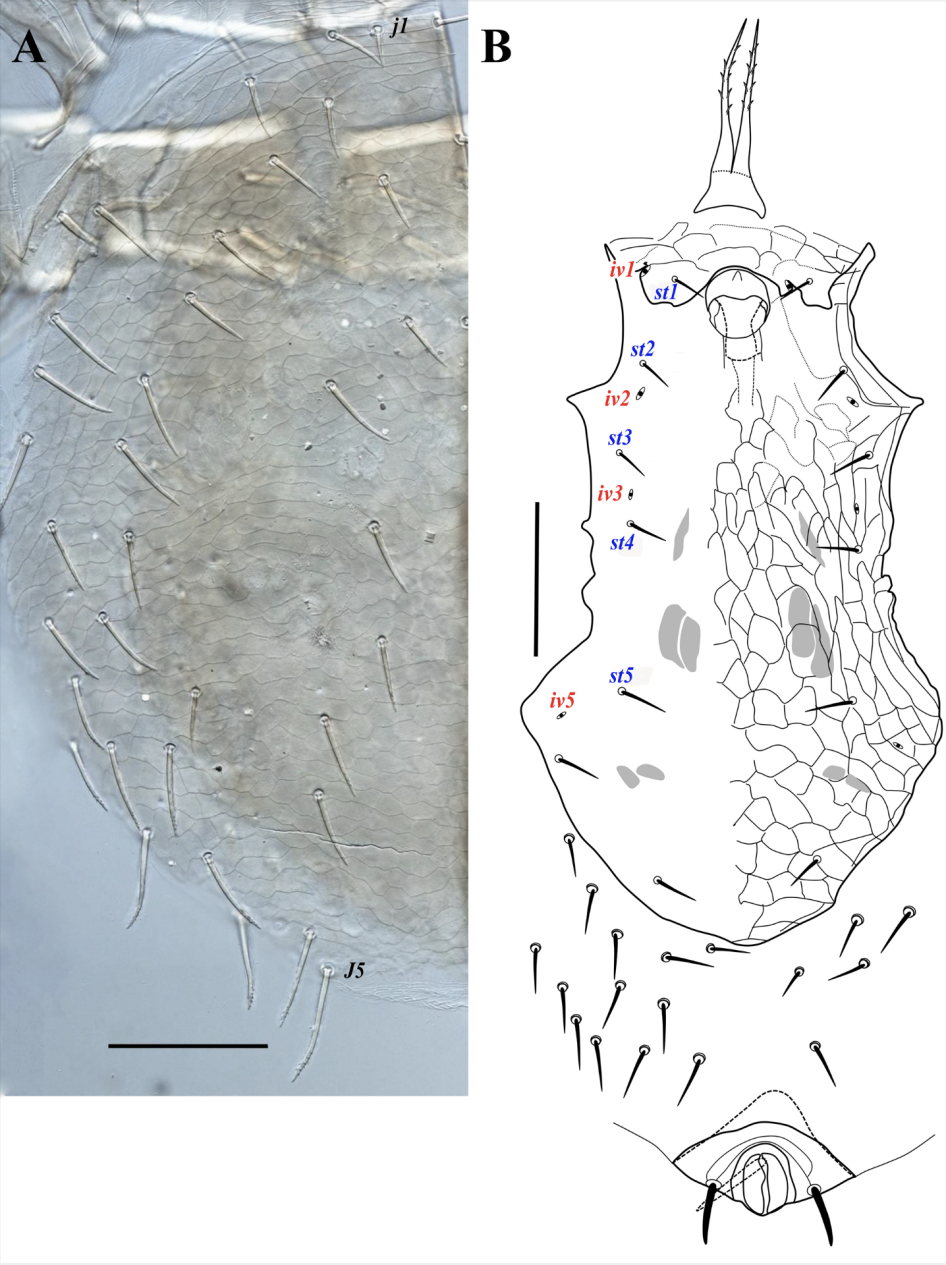
*Myrmozercon serratus* sp. nov., male. (A) Dorsal view. (B) Ventral view. Scale bar: 100 µm. Photo credit: Shahrooz Kazemi & Hans Klompen.

## Results

### *Myrmozercon serratus* sp. nov. Kazemi, Klompen, Pérez-Lachaud & Lachaud

**Table:** 

urn:lsid:zoobank.org:act:AB2A11A9-C21D-4C0F-99A7-36FD21854D8D
(Figs. 2–6, Figs.S3–S5)

#### Diagnosis

Dorsal shield with 30–34 pairs of slightly thickened and apically blunt setae, opisthonotal setae mostly with small denticles in the distal 1/3; a wide sclerotized band of marginal striations flanking the shield dorsolaterally in female, not fused, or narrowly fused medially (Figs. 2A, 2B); band bearing 6–10 pairs of setae. Female sternal shield wide, subrectangular, narrowly fused to endopodal plates between coxae III–IV laterally. Sternal setae short; setae *st4* present. Male sternitogenital shield with 6–7 pairs of setae (one ventral seta may be present or absent), *st1* on lightly sclerotized area. Epigynal shield longer than wide (length/width ratio ≈ 1.8). Anal shield with moderately enlarged anterolateral projections; circum-anal setae thickened, blunt. With 23–30 pairs of setae on dorsal soft cuticle and about 20 pairs of setae on ventral soft cuticle. Metapodal shields absent in female, present in male. Deutosternal groove with 15–16 rows of 6–25 minute denticles. Palp chaetotaxy from trochanter to genu: 1-5-6; palp tibia and tarsus fused dorsally. Cheliceral digits poorly sclerotized, edentate. Female legs trochanters I–IV with 6, 5, 5, 5, femora I–IV with 14, 11, 6, 5–7 setae, genua I–IV with 13, 11, 11, 11, and tibiae I–IV with 13, 10, 10, 10 setae, respectively; basitarsus IV with 4 or 5 setae. Male with similar leg setation, but trochanter IV with 6–7 setae; setae *al2* on genu and tibia I present or absent.

#### Description

**Table:** 

Female (N = 7)
(Figs. 2–3, 4A, 4C, 4F, 5, S3–S5)

*Dorsal idiosoma* (Figs. 2A–2C). Idiosoma length 875–930, width 695–820. Dorsal shield length 640–685, width 545–570; shield lightly sclerotized near margins, with reticulate ornamentation throughout; bearing 30–34 pairs of setae, setae may be absent asymmetrically, without unpaired *Jx* setae; setae slightly thickened, apically blunt (Fig. 2A, inset), most setae in podonotal region smooth, posteromedian setae with 0–3, and posteromarginal setae with 5–8 small denticles, opisthonotal region setae mostly with 1–10 small denticles in distal 1/3; dorsal shield setae ranging in length from 36 (*j1*) to 92 (*J3*) (Figs. 2A, S3). Shield with 14 pairs of pore-like structures, all of them resemble subcircular poroids; *id1* absent. Dorsal shield flanked by wide sclerotized bands of marginal striations, starting anteriorly at level of setae *s2*; bands not fused (Fig. 2A), or narrowly fused posteromedially (Fig. 2B); bearing 6–10 pairs of setae similar in shape to dorsal shield setae, 50–80 long. Posterior opisthonotum with soft cuticle bearing 23–30 pairs of setae, similar in shape to other dorsal setae, 50–80 long.

*Ventral idiosoma* (Figs. 3, S4). Tritosternal base columnar, short, 24–26 long, 36–42 wide at base and 24–28 at top; laciniae length 104–130 free, 11–13 fused at base, with sparse, very short barbs in distal half. Sternal shield wide, subrectangular, 135–150 long, 201–226 wide at level of setae *st2*, 290–298 at broadest width between coxae II–III; shield narrowly fused with endopodal elements between coxae III–IV; anterior margin of shield bilobed, posterior margin eroded and irregular; a narrow anterior area of shield surface with reticulate ornamentation. Sternal shield with three pairs of smooth and short sternal setae, setae shorter than half the distance between their insertions, *st1* 23–27, *st2* 28–34, *st3* 31–41 long; with two pairs of small slit-like poroids, *iv1* behind setae *st1*, *iv2* between setae *st2*–*3*. Setae *st4* (34–44 long) and adjacent poroids (*iv3*) inserted on soft cuticle. Epigynal shield longer than wide, 380–390 long, 190–230 wide at level of genital setae (*st5*), moderately wider (210–242) past setae *st5*. Setae *st5* inserted on lateral margins of epigynal shield (right margin of shield in one specimen eroded and adjacent seta *st5* off the shield), 39–45 long. Anal shield terminal; anterolateral angles of shield only slightly developed; shield 190–212 wide at broadest level, 125–155 long; circum-anal setae thick, and apically blunt, with or without a few subapical short denticles; paranal setae 52–59, postanal seta 58–65 long; cribrum well-developed, extending anteriorly to level of paranal setae. Peritremes short, extending from stigmata at mid-level of coxae IV to mid-level of coxae III. Peritrematal shields narrow, not developed posteriorly, narrowly fused to dorsal shield anteriorly, pore-like structures on the shields not observed. Opisthosomal soft cuticle usually with one pair of narrow and short platelets flanking posterior margins of epigynal shield, sometimes with closer platelets partially fused with the shield, rarely without platelets; parapodal plates narrow; metapodal plates absent; seven pairs of poroids, including *iv5* and *ivp*, and about 20 pairs of setae; 8-10 pairs of pre-anal setae smooth, marginal setae longer, thicker, and denticulate (similar to dorsal shield setae); length ranging from 31 to 77. Insemination ducts opening on posterior margin of coxae III.

*Gnathosoma* (Figs. 2D, 4A, 4C, 4F, S5). Gnathotectum subtriangular, with smooth lateral margins and regular or irregular rounded tip. Supralabral process short and undivided apically, without apicoventral projection (Fig. 2D). Hypostome with paired internal malae with moderately narrow anterior projections, with smooth margins, bearing 3–5 small narrow denticles on posterolateral margins, almost as long as corniculi. Corniculi membranous, lightly sclerotized laterally (Fig. 4A). Labrum blade-like, with short and dense fringed margins, anteriorly extending almost to level of corniculi. Hypostomal setae smooth, pointed, *h1* 22–26, *h2* 24–26, *h3* 39–44, *sc* 28–30 long. Deutosternal groove moderately wide, lateral ridges flanking 15–16 rows of about 10 (basal rows) to 25 minute denticles, and an anterior smooth ridge. Second segment of chelicera narrow, 163–166 long; cheliceral digits edentate, fixed digit 50–54 long, pilus dentilis vestigial; movable digit 75–77 long, partially membranous; dorsal cheliceral seta, dorsal poroid and slit-like lateral poroid present (Fig. 4C). Palp 161–170 long, palp chaetotaxy from trochanter to genu: 1-5-6, tibia and tarsus fused dorsally, palp apotele two tined (Fig. 4F).

*Legs* (Fig. 5). All legs with well-developed ambulacra, pulvilli large, claws not developed, pretarsal operculae well-developed, two-tined apically (Figs. 5B–5D, inset). Lengths of legs I –IV: 1135–1160, 710–740, 825–860, and 990–1035, respectively. Leg chaetotaxy as in Table 1; femur IV with 5, 6, or 7 setae (one seta lost or added relative to the “standard” pattern for Laelapidae), basitarsus IV occasionally with an additional seta bringing the total number to 5 (both states observed in one specimen) (Fig. 5D). Leg setae smooth, setae on coxae I–IV and several setae on trochanters and tarsi I–IV pointed, other setae mostly blunt; setae *ad* and *av* on trochanter I, setae *ad1*–*2*, *pd1*, *av1*–*2*, *pv2*–*3* on femur I, setae *ad1* and *pd1* on femur II, setae *pd* and *ad* on trochanter III, setae *ad*, *pd1*–*2*, and seta *al* on femur III, setae *al* and *ad* on trochanter IV, setae *al*, *ad1*–*2*, *av*, *pd1* on femur IV thickened; several setae on genu and tibia I, genu II, genua and tibiae III–IV slightly thickened; legs without elongate seta.

**Table:** 

Male (*N* = 1)
(Fig. 4D, 6)

*Dorsal idiosoma* (Fig. 6A). Dorsal shield length 597, width 545, bearing 34 pairs of setae, *j1* shortest (28), *J5* longest (74), length of other setae 38–71. Dorsal soft cuticle with 18–22 pairs of setae. Wide sclerotized band of marginal striations absent. Other characters similar to female.

**Table 1:** Leg chaetotaxy of *Myrmozercon serratus* sp. nov. female (N = 5).

**Leg**	**Coxa**	**Trochanter**	**Femur**	**Genu**	**Tibia**	**Tarsus**
I	2	6	14 (2 3/2 2/3 2)	13 (2 3/2 3/1 2)	13 (2 3/2 3/1 2)	–
II	2	5	11 (2 3/1 2/2 1)	11 (2 3/1 2/1 2)	10 (2 2/1 2/1 2)	4 + 14
III	2	5	6 (1 1/0 2/0 2)	11 (2 2/1 3/1 2)	10 (2 1/1 3/1 2)	4 + 14
IV	1	5	5, 6 or 7	11 (2 2/1 3/1 2)	10 (2 1/1 3/1 2)	(4 or 5) + 14

**Notes.**

Formulas following Evans (1963a).

*Ventral idiosoma* (Fig. 6B). Tritosternal base 21 long, 35 wide at base and 23 wide at apex, laciniae 101 free and fused for 6 µm. Sternitogenital shield length 411, width 163 at level of *st2*, 274 at broadest level behind coxae IV; anterior area of shield lightly sclerotized, bearing setae *st1*, 27 long, apically pointed; well-sclerotized area of shield with reticulate surface ornamentation, bearing *st2*–*5* (27–31 long), and three ventral setae: two on left and one on right side of shield, 24 –30 long, setae *st1*–*4* apically pointed, others blunt; shield with four pairs of poroids, *iv1*–*3* small and slit-like, *iv5* round, *iv1* on anterior margin of well-sclerotized area of shield. Anal shield terminal, length 121, width 167, circum-anal setae thickened, apically rounded; postanal seta (47) slightly longer than paranals (43). Opisthogastral soft cuticle with a pair of lightly sclerotized metapodal shields, and about 24 pairs of blunt setae; posterolateral setae with small denticles in distal 1/3.

*Gnathosoma*. Hypostomal setae length: *h1* 21, *h2* 20, *h3* 35, *sc* 27. Deutosternal groove with 16 rows of multiple small denticles, and an anterior smooth ridge. Second segment of chelicera 157 long; cheliceral digits edentate, fixed digit lightly sclerotized, 56 long; movable digit, including straight spermatodactyl, well sclerotized, 120 long (Fig. 4D). Palp 155 long. Other structures similar to female.

*Legs*. Length of legs I–IV 990, 593, 733 and 855, and pretarsi I–IV 77, 62, 63 and 65, respectively. Pretarsal claws I–IV not developed, but pulvilli of all legs, and pretarsal operculae II–IV (apically divided into two tines) well-developed. Chaetotaxy as in female (Table 1) with the following exceptions: left trochanter IV with 7 and right segment with 6 setae; seta *al2* on genu and tibia I absent on one side of the body, present on the other; right basitarsus IV with 4 setae, left one with 5. Setal shapes as in female.

**Table:** 

Deutonymph (*N* = 3)
(Figs. 4B, 4E)

*Dorsal idiosoma.* Dorsal shield length 590, width 450, shield lightly sclerotized on margins; with 32 pairs of slightly thickened setae, setae smooth or with small denticles in apical third, blunt; length of setae ranging from 20–56, *j1* shortest and *Z5* longest. Wide sclerotized band of marginal striations absent. Other characters similar to female.

*Ventral idiosoma.* Tritosternal base 16 long, 28 wide at base and 22 at apex, laciniae 84 free and fused for 22 µm. Sternal shield lightly sclerotized, with faint reticulate ornamentation, margins of shield not distinct, setae *st1*–*3* (24–28) and poroids *iv1*–*3* on moderately sclerotized margins of shield, *st4* (24) and *st5* (20) and *iv4* on unsclerotized margins of shield. Anal shield terminal, lightly sclerotized, anterolateral projections of shield not developed. Other characters as in female.

*Gnathosoma* (Figs. 4B, 4E) Hypostomal setae length: *h1* 24, *h2* 20–21, *h3* 36–37, *sc* 27. Deutosternal groove with 16 rows of denticles, basal row with 2 denticles, second row with 6, third row with 9, and others with about 12 to 25 smaller denticles, median rows wider, with an anterior smooth ridge (Fig. 4B). Second segment of chelicera 150 long; cheliceral digits edentate, movable and fixed digits 66 and 46 long, respectively; arthrodial brush with a row of lateral denticles (Fig. 4E). Palp 168 long. Other structures as in female.

*Legs*. Lengths of legs I–IV: 848, 595, 718, and 824, respectively. Leg chaetotaxy as in female (Table 1) with the following exceptions: femora IV with 6 setae (no variability); basitarsi IV with 4 setae (no variability). Setal shapes as in female.

#### Larva

This instar was collected but the available specimen was in poor condition and is therefore not described.

#### Type depository

Holotype female deposited at Colección Nacional de Ácaros, Instituto de Biología, UNAM, Mexico (CNAC 012527). Paratypes at OSAL and ECO-CH-AR.

#### Material examined

Mexico, Quintana Roo, Laguna Guerrero, 8 m asl [18.6920, -88.2615], coll. Pérez-Lachaud, G., 1-Aug-2021, in a colony of *C. atriceps* (Hymenoptera: Formicidae) nesting in a dead branch of a *Hibiscus* shrub, 1-F, holotype, CNAC 012527; same locality, collector, collection date, and source, 1-F, OSAL 159521; 1-F, OSAL 159522; 1-F, OSAL 159523; 1-M, OSAL 159520; 1-DN, OSAL 159525; same host species, locality and collector, 17-Jul-2022, in a trap-nest, 3-F 1-M, in 75% ethanol, OSAL 160580; 15-Jan-2022, same host species, locality and collector, nest in hanging dry branch, 2-F, in 75% ethanol, OSAL 160581; 10-Aug-2022, in a bamboo trap-nest, 2-F 1-DN, in 75% ethanol, OSAL 160582; same host species, locality and collector, 15-Aug-2021, in a trap-nest, 3 adults of unknown sex, ECO-CH-AR AA3479-AA3481.

Additional material: Mexico, Chiapas, Huixtla, 52 m asl, coll. Lachaud, J.-P., 6-Sep-2005, in a colony of *C. atriceps* in mango plantation, 2-F, OSAL 161616–161617.

#### Etymology

The species epithet “*serratus*” was chosen on the basis of the shape of most setae in the opisthonotal region of the dorsal shield.

#### Notes on the species

With a combination of characters, including the long legs with hypertrichous setae, the reticulate dorsal shield, and the subrectangular sternal shield, *M. serratus* sp. nov. is a member of the species group designated *Myrmozercon sensu lato* by Joharchi, Babaeian & Seeman (2015). Within this group, the newly described species is most similar to *M. antennophoroides* (Berlese) (Berlese, 1903), *M. hunteri* Joharchi, Babaeian & Seeman (Joharchi, Babaeian & Seeman, 2015), and *M. patagonicus* Trach & Khaustov (Trach & Khaustov, 2018) by the presence of 30–34 pairs of setae on the dorsal shield and a subrectangular sternal shield. *Myrmozercon serratus* sp. nov. differs from *M. antennophoroides* by the presence of only one pair of setae on the epigynal shield (two pairs in *M. antennophoroides*), legs I much longer than dorsal shield, their length ratio ≈ 1.7 (legs I shorter in *M. antennophoroides*, legs I/dorsal shield length ratio ≈ 1.37), and gnathotectum subtriangular, but no elongate in the new species (subtriangular and elongate in *M. antennophoroides*). The new species can be distinguished from *M. hunteri* by the presence of 6 setae on trochanter I and 13 setae on tibiae I (5 and 10 setae, respectively, in *M. hunteri*), presence of simple setae with pointed tips on the legs (most setae on legs with club-like tip in *M. hunteri*), and presence of a wide sclerotized band of marginal striations surrounding the dorsal shield (absent in *M. hunteri*). *Myrmozercon patagonicus* can be differentiated from *M. serratus* sp. nov. by the presence of 19–21 rows of denticles in the deutosternal groove (15–16 rows in *M. serratus* sp. nov.), presence of metapodal platelets (absent in *M. serratus* sp. nov.), 4 setae on palp femur (5 in the new species), and also 14 setae on genua I, 7–8 setae on femora III and 8 setae on femora IV (13, 6, and 6 setae, respectively, in *M. serratus* sp. nov.).

**Figure 7:**
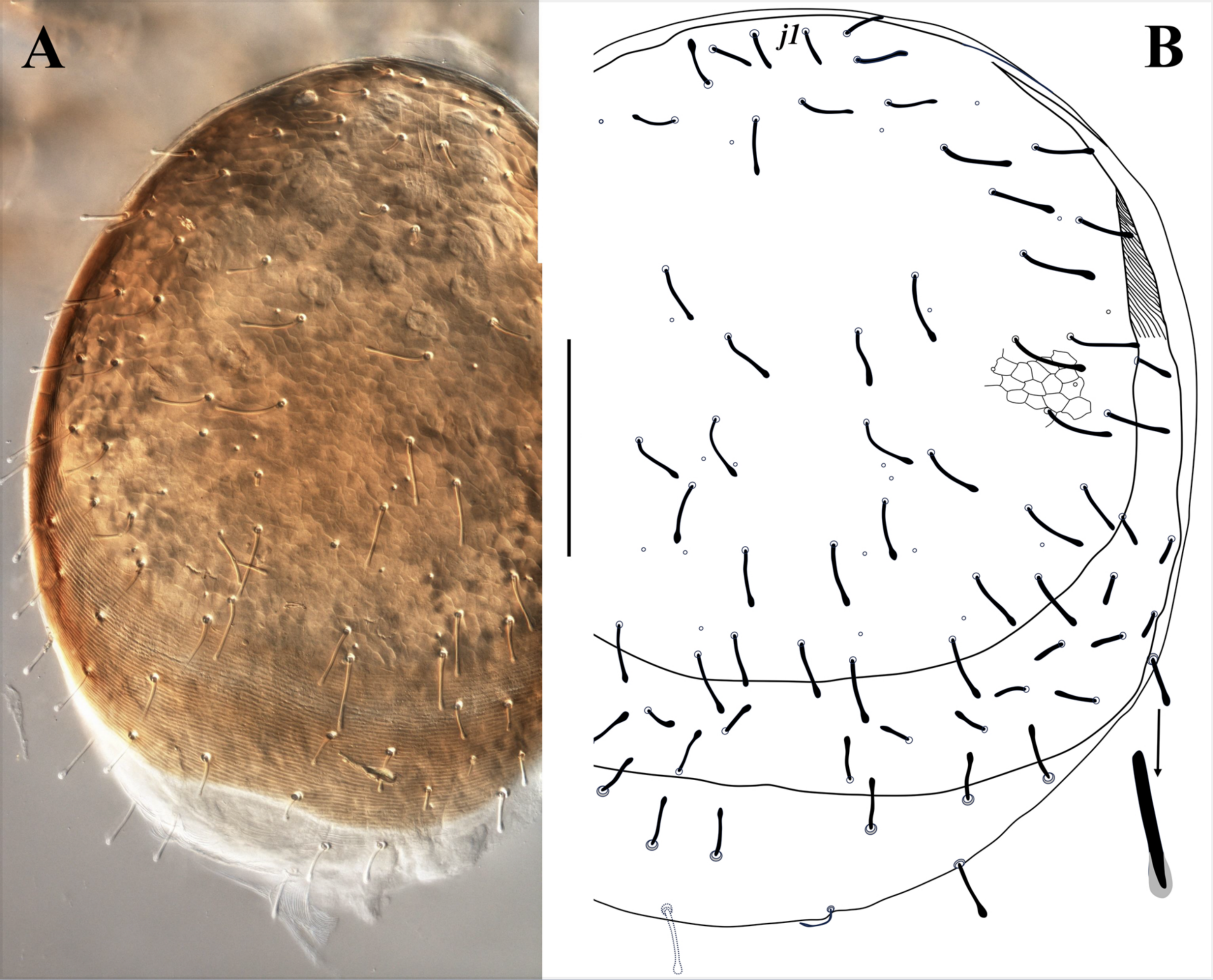
*Myrmozercon spatulatus* sp. nov., female. (A, B) Dorsal views. Scale bar: 200 µm. Photo credit: Shahrooz Kazemi & Hans Klompen.

**Figure 8:**
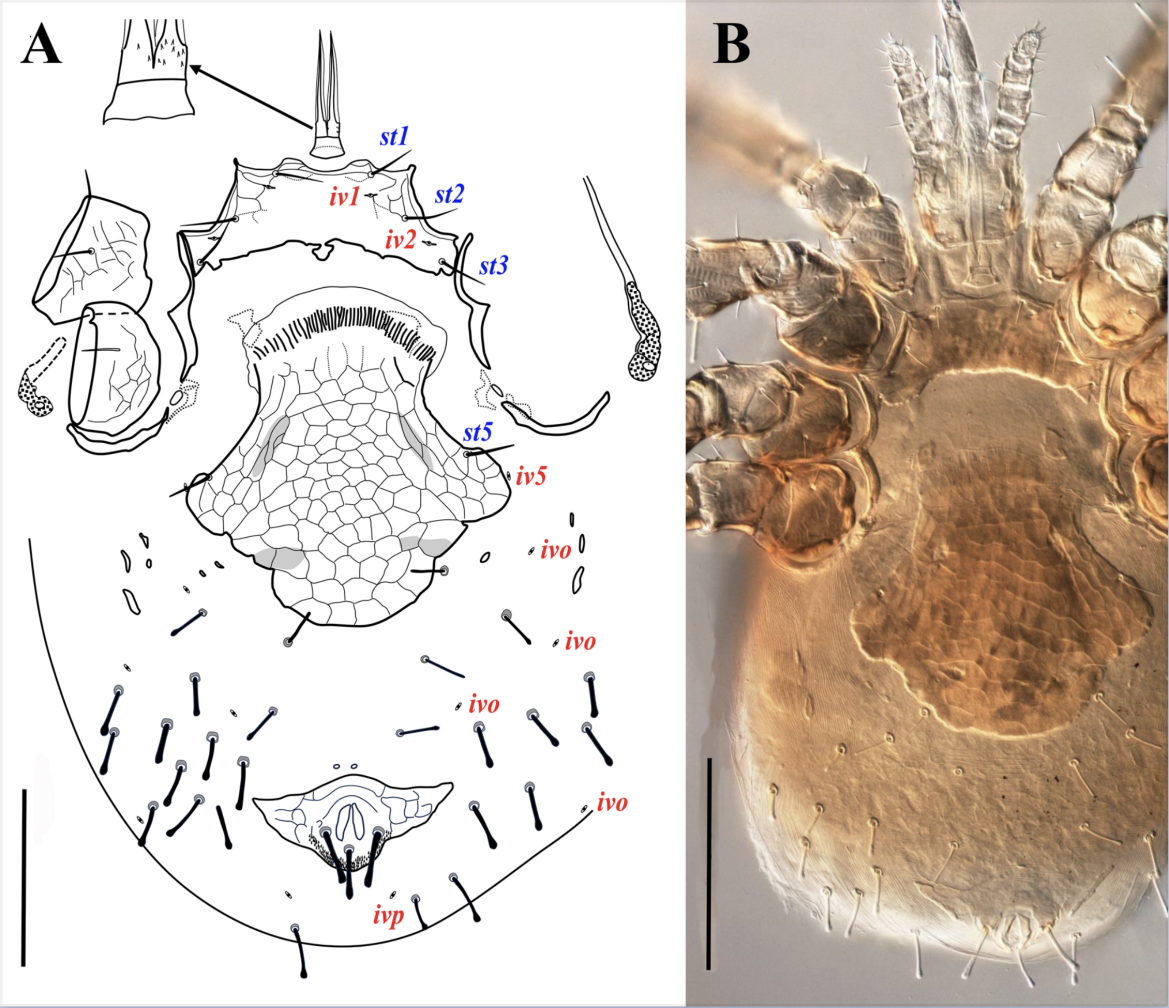
*Myrmozercon spatulatus* sp. nov., female. (A, B) Ventral views. Scale bars: 200 µm. Photo credit: Shahrooz Kazemi & Hans Klompen.

**Figure 9:**
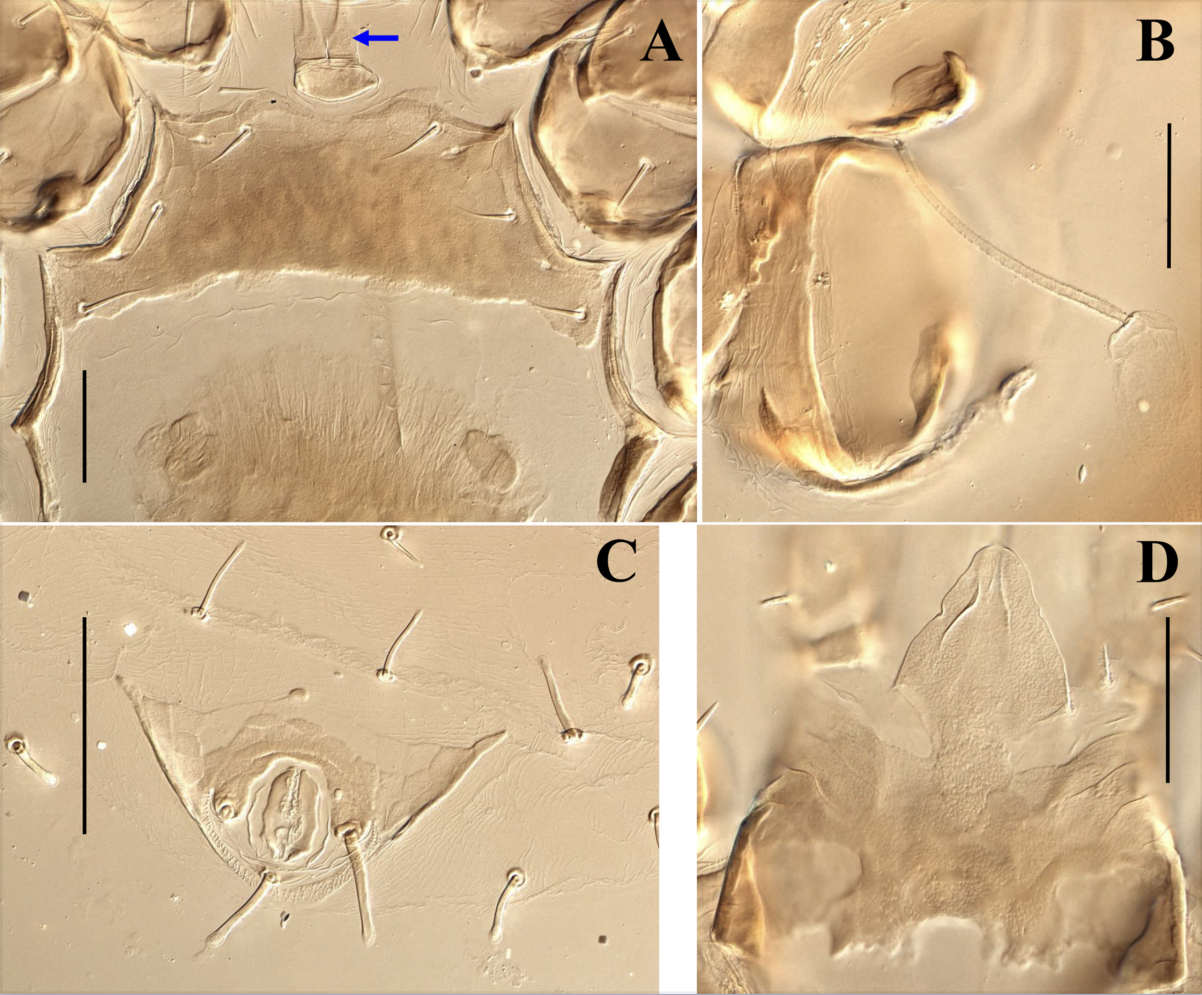
*Myrmozercon spatulatus* sp. nov., female, details. (A) Sternal shield and tritosternum (arrow: ventral spinelets on base tritosternum). (B) Bursa copulatrix. (C) Anal shield. (D) Gnathotectum. Scale bars: 50 µm. Photos credit: Shahrooz Kazemi & Hans Klompen.

**Figure 10:**
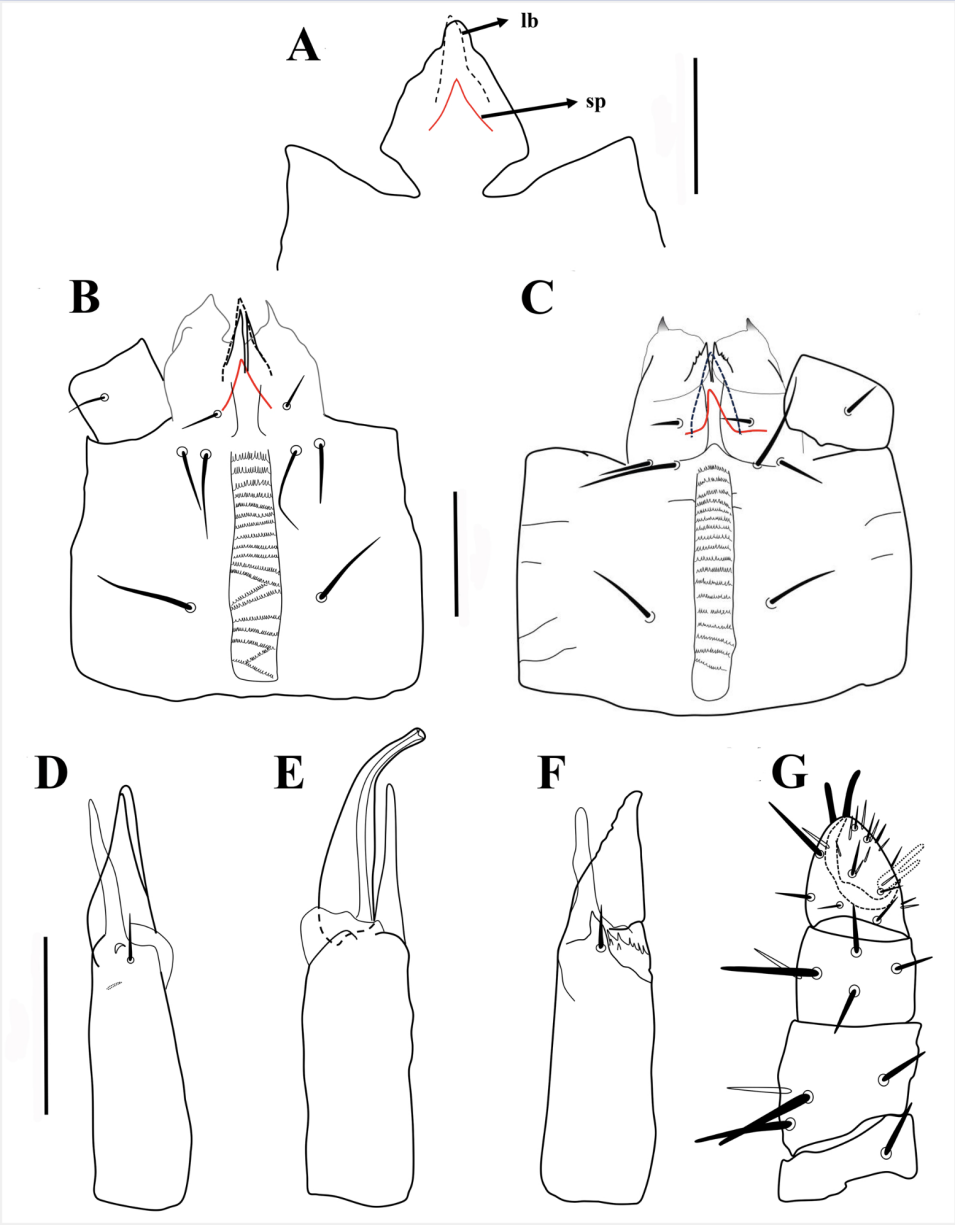
*Myrmozercon spatulatus* sp. nov. (A) Gnathotectum female. (B) Subcapitulum female. (C) Subcapitulum deutonymph. (D) Chelicera female. (E) Chelicera male. (F) Chelicera deutonymph. (G) Palp female. Scale bars: 50 µm; abbreviations: *lb* = labrum; *sp* = supralabral process.

**Figure 11:**
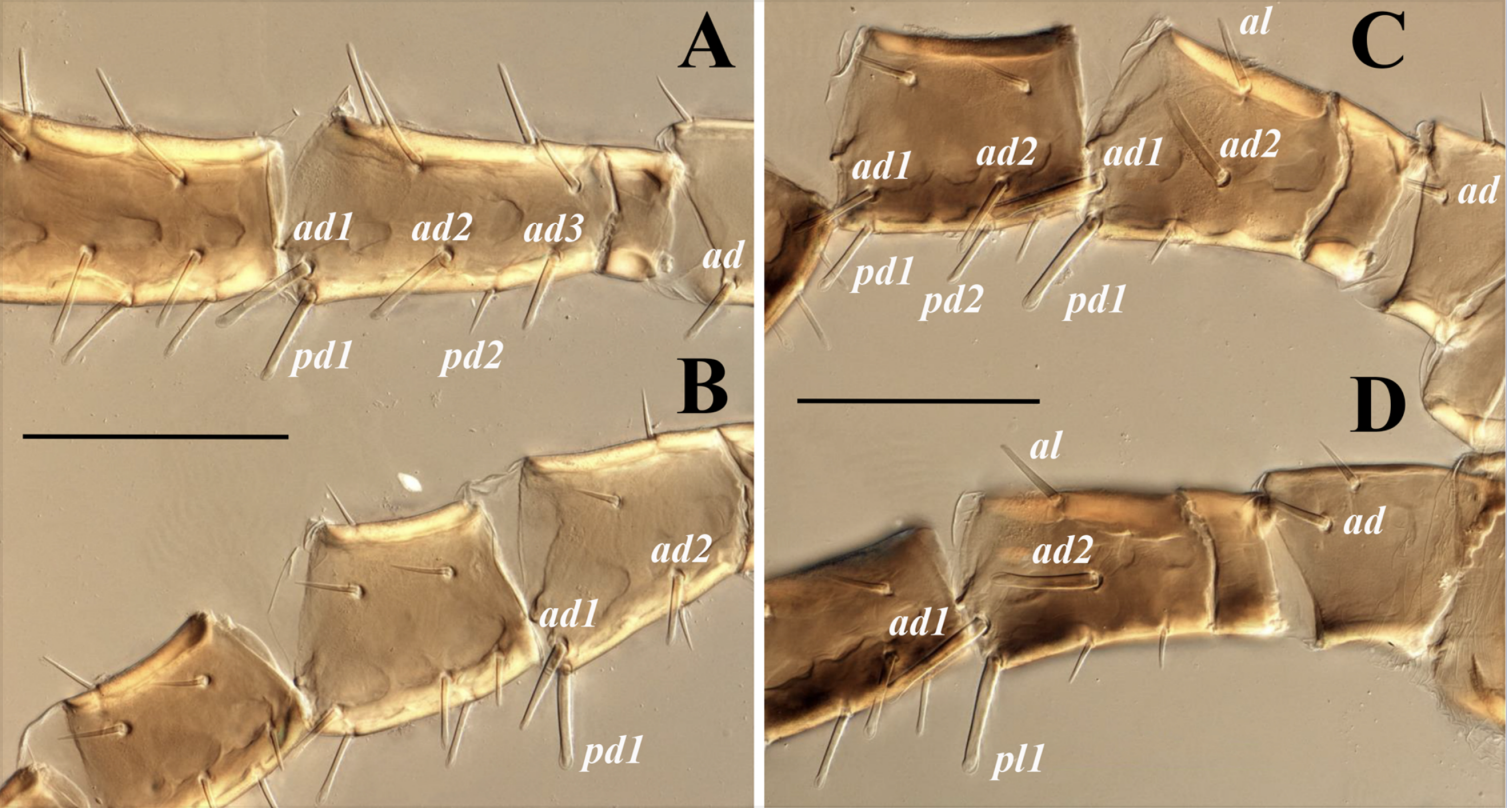
*Myrmozercon spatulatus.* sp. nov., female. (A–D) Partial view of femora and genua of legs I–IV. Scale bars: 200 µm. Photos credit: Shahrooz Kazemi & Hans Klompen.

**Figure 12:**
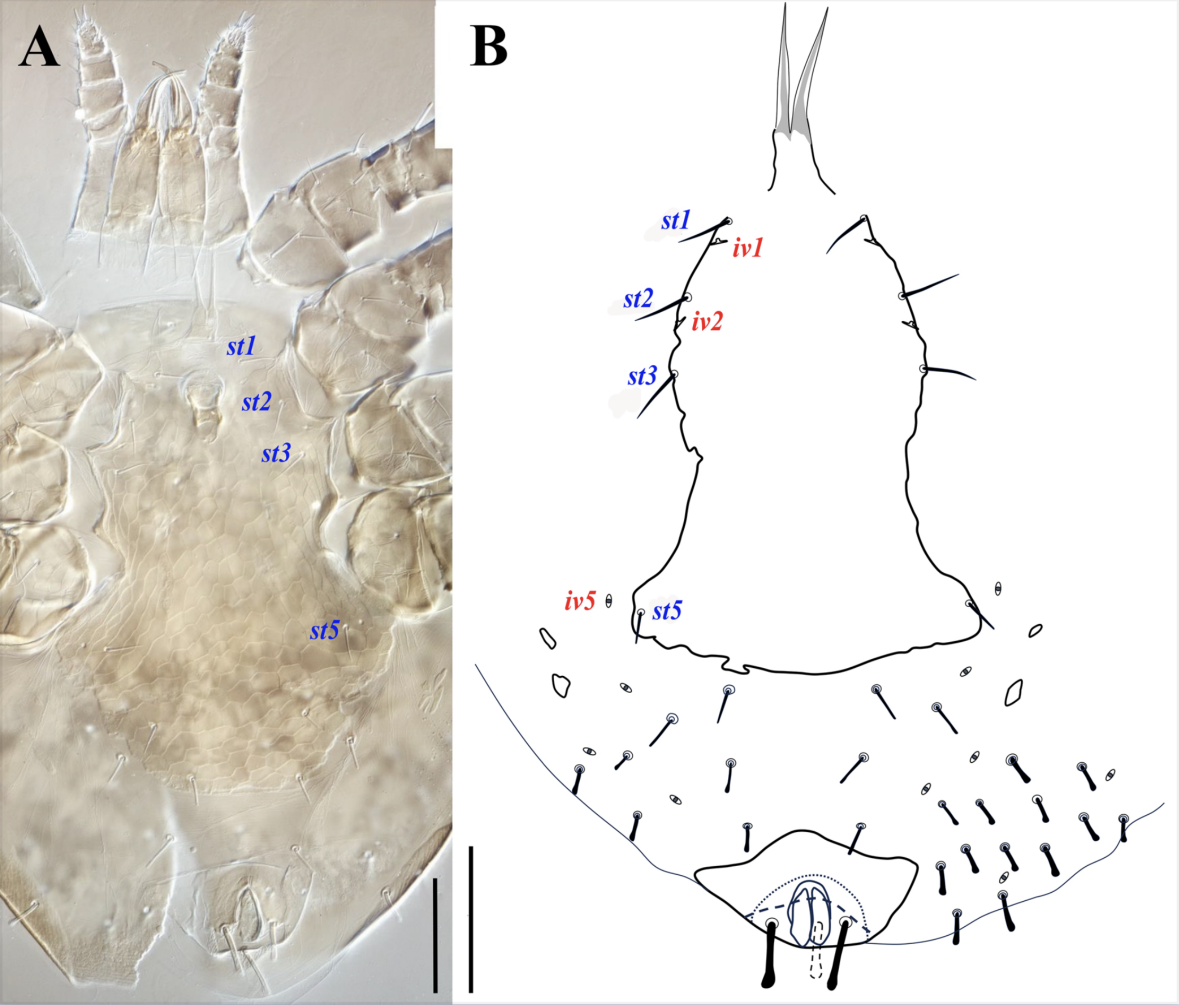
*Myrmozercon spatulatus* sp. nov. (A) Male, ventral view. (B) Deutonymph, ventral view. Scale bars: 100 µm. Photo credit: Shahrooz Kazemi & Hans Klompen.

### *Myrmozercon spatulatus* sp. nov. Kazemi, Klompen, Pérez-Lachaud & Lachaud

**Table:** 

urn:lsid:zoobank.org:act:50BA702F-CCB1-488D-A3AC-0618B9F5881A
(Figs. 7–12)

#### Diagnosis

Dorsal shield with 27–31 pairs of slightly thickened and apically spatulate setae; wide sclerotized band of marginal striations well-developed in female (less developed in male), surrounding the shield laterally and broadly fused posteromedially; band bearing 10–12 pairs of setae. Female sternal shield wide, moderately narrow; with relatively long sternal setae, *st2*–*3* as long as the distance between their insertions, *st1* slightly shorter than *st1*–*2* interval. Female sternal shield adjacent to, but not fused to, endopodals between coxae III–IV. Setae *st4* absent. Male sternitogenital and deutonymphal sternal shield bear five and four pairs of setae, respectively. Epigynal shield large and wide, almost as long as wide (length/width ratio ≈ 0.9). Anal shield wide, anterolateral edges well-developed, circum-anal setae thickened, apically spatulate. With 7–10 pairs of setae on dorsal and 14–16 pairs of setae on ventral soft cuticle. Metapodal shields present. Deutosternal groove with 18–19 rows in female and deutonymph, and 15 rows in male, each row with multiple denticles; palp chaetotaxy from trochanter to genu: 1-4-5. Cheliceral digits poorly sclerotized, edentate; spermatodactyl sickle-shaped. Female femora I–IV with 13, 10, 6–7, 8–9 setae, genua I–IV with 13, 11, 11, 11, and tibiae I–IV with 13, 10, 10, 10 setae, respectively. Leg setae, especially dorsal setae on trochanter to tibia, apically spatulate.

#### Description

**Table:** 

Female (*N* = 11)
(Figs. 7–9, 10A, 10B, 10D, 10G, 11)

*Dorsal idiosoma* (Fig. 7). Idiosoma 645–770 long, 590–620 wide. Dorsal shield with a reticulate ornamentation throughout; 570–650 long, 535–555 wide; bearing 27–31 pairs of setae, setae *j1* shortest (26–28), slightly thickened with blunt tip, other setae 29–58 long, thickened, with spatulate tip (Fig. 7B, inset), mostly smooth, rarely with small lateral denticles behind spatulate tip. Dorsal shield with 10 pairs of pore-like structures, *id1* absent. Dorsal shield flanked with well-developed sclerotized band of marginal striations, bearing 10–12 pairs of setae similar in shape to those on dorsal shield, 35–45 long. Posterolateral membranous area of dorsal idiosoma with 7–10 pairs of setae, similar in length and shape to setae on sclerotized band of marginal striations.

*Ventral idiosoma* (Figs. 8, 9A–9C). Tritosternum with a short columnar base, 31–33 long, 35–39 wide at base and 26–28 wide at apex; laciniae free, total length 88–99, sclerotized near the base for 12–14 µm, with 1–12 minute ventral spicules and smooth hyaline margins (Figs. 8A, 9A, arrows). Sternal shield wide, 70–85 long, 187–194 wide at level of setae *st2*, 254–256 at greatest width between coxae II–III; anterior margin bilobed, posterior margin eroded, irregularly concave, sometimes with a small median projection, up to 15 long; posterolateral margins of shield adjacent to, but not fused to, endopodal elements between coxae III–IV. Anterior and anterolateral regions of sternal shield with reticulate ornamentation, median reticulation faint. Sternal shield with three pairs of smooth and moderately long sternal setae, *st1* 43–47, *st2*–*3* 45–48 long, and two pairs of slit-like poroids. Setae *st4* and associated poroids (*iv3*) absent. Epigynal shield wide, expanded behind coxae IV, with irregularly convex posterior margin; surface reticulate; 278–286 long, 315–319 wide at broadest level beyond setae *st5* insertion; setae *st5* on lateral margins of shield, 36–46 long. Anal shield with well-developed anterolateral projections; anterior margin finely convex and lightly sclerotized; 95–115 long and 205–220 wide at broadest level; cribrum well-developed; circum-anal setae thick, and apically spatulate, paranal setae (52–57) slightly longer than postanal (48–51) (Fig. 9C). Peritrematal shields narrow, not developed beyond stigmata, narrowly extended anteriorly and fused to dorsal shield; pore-like structures invisible in specimens examined. Peritremes short, extending anteriorly from stigmata at posterior level of coxae IV to anterior level of coxae IV or posterior level of coxae III. Opisthogastral soft cuticle with one pair of narrow parapodal plates, 1–3 pairs of moderately small and narrow metapodal platelets, 1–2 minute paragenital platelets lateral to *Jv1*; with 14–16 pairs of setae, *Jv1* (30–35) moderately slender with blunt apex, *Jv2*–*4* and *Zv2* slightly thickened (32–42), others thickened with spatulate tip (32–55); with six pairs of poroids, including *iv5* (behind *st5* level), *ivp* and four pairs of *ivo* (Fig. 8A). Insemination ducts opening on posterior margin of coxae III (Fig. 9B).

*Gnathosoma* (Figs. 9D, 10A, 10B, 10D, 10G). Gnathotectum subtriangular, with smooth lateral margins and regular or irregular rounded tip, connected to dorsum of gnathosoma by a narrow neck (Figs. 9D, 10A). Supralabral process subtriangular, short (Fig. 10A, *sp*); labrum blade-like, with fringed margins, extending slightly beyond anterior margin of gnathotectum (Fig. 10A, *lb*). Hypostome with paired internal malae with moderately narrow and smooth anterior projections, not extending to anterior level of corniculi; corniculi membranous. Hypostomal setae smooth, *h1* 11–14, *h2* 21–23, *h3* 38–41, *sc* 31–33. Deutosternal groove wide, lateral ridges flanking 18–19 rows of multiple denticles, arranged between smooth anterior and posterior ridges; denticles in most rows subequal in size, except in anteriormost row which has larger denticles (Fig. 10B). Second segment of chelicera narrow, 113–116 long; cheliceral digits edentate, fixed digit 36–38 long, pilus dentilis vestigial; movable digit 51–53 long, partially membranous; dorsal cheliceral seta, dorsal and lateral poroids present (Fig. 10D). Palp 104–108 long, palp chaetotaxy from trochanter to genu: 1-4-5, tibia and tarsus fused dorsally, with 23 setae, palp tarsus apotele two-tined (Fig. 10G).

*Legs* (Fig. 11). All legs with pretarsus and well-developed ambulacrum, pretarsal operculae II–IV well developed, divided into 4–5 apical branches. Lengths of legs I–IV: 780–800, 555–570, 615–635, 660–675. Leg chaetotaxy as in Table 2. Leg setae smooth, setae on coxae I–IV and several setae on trochanters and telotarsi I–IV slender and pointed, most other leg setae apically blunt or spatulate, slender or slightly thickened, except setae *ad* on trochanter I, setae *ad1*–*3* and *pd1* on femur I (Fig. 11A), setae *ad1*–*2* and *ad1* on femur II (Fig. 11B), setae *al*, *ad1*–*2* and *pd1* on femur III (Fig. 11C), and setae *al*, *ad1*–*2* and *pd1* on femur IV (Fig. 11D) which are thickened with spatulate tips; legs without elongate setae.

**Table:** 

Male(N = 2)
(Figs. 10E, 12A)

*Dorsal idiosoma*. Idiosoma of well sclerotized specimen 649 long, 530 wide, that of less sclerotized male 550 long, 477 wide. Dorsal shield with reticulate ornamentation throughout; length and width of shield in well-sclerotized specimen 587 and 530, respectively, not measured in less sclerotized specimen because shield margins were not detected; shield bearing 32 (26 in less sclerotized specimen) pairs of setae, 20–42 long, with 14 pairs of pore-like structures. Wide sclerotized band of marginal striations distinct in well-sclerotized specimen, with two pairs of setae, 32–35 long. Opisthosomal soft cuticle in well-sclerotized specimen devoid of setae, with four pairs of setae in less sclerotized specimen. Other characters similar to female.

**Table 2:** Leg chaetotaxy of *Myrmozercon spatulatus* sp. nov., female (N = 5).

**Leg**	**Coxa**	**Trochanter**	**Femur**	**Genu**	**Tibia**	**Tarsus**
I	2	6	13 (2 3/2 2/2 2)	13 (2 3/2 3/1 2)	13 (2 3/2 3/1 2)	–
II	2	5	10 (2 2/1 2/2 1)	11 (2 3/1 2/1 2)	10 (2 2/1 2/1 2)	4 + 14
III	2	5	6, 7	11 (2 2/1 3/1 2)	10 (2 1/1 3/1 2)	4 + 14
IV	1	5	8 (1 2/2 3/0 0) 9 on 1 segment	11 (2 2/1 3/1 2)	10 (2 1/1 3/1 2)	4 + 14

**Notes.**

Formulas following Evans (1963a).

*Ventral idiosoma* (Fig. 12A). Tritosternal base 10–12 long, 28–30 wide at base and 22 wide at apex; laciniae free, total length 80–86, sclerotized near the base for 10–12 µm, without ventral minute spicules, other characters similar to female. Sternitogenital shield wide, extending well beyond coxae IV, widened behind coxae IV; reticulate over entire surface, area between tritosternal base and anterior margin of well-sclerotized region of sternitogenital shield poorly sclerotized, bearing setae *st1*; posterior margin of shield irregularly convex, shield 355–381 long, 165–180 wide at level of setae *st2*, 283–303 wide at broadest level beyond coxae IV. Setae *st1*–*3* slender, with pointed apex (whip-like), 34–37 long, *st5* with rounded tip (26–33), *Jv1* and *Zv1* (19–22) with lightly spatulate tip; with three pairs of poroids, *iv1*–*2* small and slit-like, *iv5* round, *iv3* absent. Anal shield with slightly convex anterior margin and anterolateral projections, 102–105 long and 162–180 wide at broadest level; paranal setae 35–43, postanal 43–48 long. Opisthogastral soft cuticle with 3–5 narrow metapodal platelets; cuticle bearing 3–5 pairs of setae (26–30) with spatulate apex and 5 pairs of poroids.

*Gnathosoma* (Fig. 10E). Gnathotectum similar to female. Hypostome, corniculi, labrum and supralabral process as in female. Hypostomal setae *h1* 10–11, *h2* 15–18, *h3* 32–38, *sc* 23–27 long. Deutosternal groove wide, with 16 rows of multiple small denticles. Second segment of chelicera narrow, 120–128 long; cheliceral digits edentate, fixed digit 41–46 long, movable digit, including sickle-shaped spermatodactyl, 78–81 long (Fig. 10E). Palp 110–114 long, palp chaetotaxy similar to female. Other characters as in female.

*Legs*. Lengths of legs I–IV: 700–710, 485–515, 545–560, 575–605. Leg chaetotaxy as in female (Table 2) with the following exceptions: tibia I occasionally lacking seta *av2*; femur II on one side of one specimen with only 9 (instead of 10) setae; tibia III occasionally with an extra seta (11 instead of 10 total); femur IV never with ninth seta. Setal shapes as in female.

**Table:** 

Deutonymph (*N* = 2)
(Figs. 10C, 10F, 12B)

*Dorsal idiosoma.* Idiosoma 588 long, 498 wide. Dorsal shield 495 long, 431 wide, with a reticulate ornamentation throughout; bearing 28 pairs of thickened setae with spatulate apex, setae mostly smooth, rarely with lateral small denticles (*e.g.*, in *J4*, *J5*, and *Z5*); 24–38 long, *j1* shortest, *Z5* longest. Wide sclerotized band of marginal striations absent. With about 15–20 pairs of setae on opisthosomal soft cuticle, similar in shape to those on dorsal shield, 19–28 long. Other characters similar to female.

*Ventral idiosoma* (Fig. 12B). Tritosternal base 10 long, 30 wide at base and 22 at apex; laciniae with smooth membranous margins, 95–99 free, 6 fused at base, without ventral minute spicules. Sternal shield wide, with reticulate surface ornamentation; 316 long, 152 wide at level of setae *st2*, 241 at broadest level behind coxae IV. Sternal shield with four pairs of smooth setae, *st1* 36, *st2*–*3* 39 long, with pointed apex, *st5* 20 long, blunt; with three pairs of small slit-like poroids *iv1*–*2*, *iv5*. Posterior margin of sternal shield irregularly convex. Anal shield terminal, with anterolateral projections, 120 long, 167 wide at broadest level; circum-anal setae thick and apically spatulate, paranal setae 44 and 47 and postanal seta 38 long. Opisthogastral soft cuticle with two pairs of small metapodal platelets, seven pairs of poroids (including *iv5*, *ivp*, *idR3* and four pairs of *ivo*), and 14–18 pairs of setae: *Jv1* (26) narrow and blunt, *Jv2* (23), *Jv3* (22), and *Zv1* (26) slightly thickened with spatulate apex, 10–14 additional pairs of thickened and moderately short (20–26) setae with spatulate apex.

*Gnathosoma* (Figs. 10C, 10F). Gnathotectum subtriangular, with irregular and smooth anterior margin and rounded apex. Hypostome, internal malae, labrum and supralabral process similar to female (Fig. 10C). Corniculi wide, mainly membranous, but with short horn-like sclerotized tips. Hypostomal setae smooth, sharp, *h1* 11, *h2* 18, *h3* 40, *sc* 28 long. Deutosternal groove moderately wide, lateral ridges flanking 18 rows of multiple small denticles, each row with 12–18 denticles, with smooth anterior (inverse V-shaped) and posterior (concave) ridges. Cheliceral digits poorly sclerotized, edentate, second segment of chelicera 116 long, fixed digit 35 long, movable digit 52 long, arthrodial brush with a row of denticles, extending laterally to venter, ventral denticles largest; dorsal cheliceral seta well developed (Fig. 10F). Palp 104 long, palp chaetotaxy from trochanter to tarsus: 1-4-5, palp apotele two-tined. Other characters similar to female.

*Legs*. Lengths of legs I–IV: 710, 526, 579, 612. Leg chaetotaxy as in female (Table 2) with the following exceptions: right leg tibia I with 14, not 13, setae (*pl3* added); both legs with 7 setae on femora II (not 6 or 7); basitarsus IV of one leg with 5 (not 4) setae. Setal shapes as in female.

#### Larva

This instar was collected but specimens were in poor condition and only a few aspects can be described. Except for sternal (10–18 long, needle-like), circum-anal setae (paranals 8–9, postanal 12 long, cone-like), and most dorsal setae on telotarsus I, all remaining setae minute (3–5) or vestigial. No distinct shields observed.

#### Type depository

Holotype female deposited at Colección Nacional de Ácaros, Instituto de Biología, UNAM, Mexico (CNAC 012528). Paratypes at OSAL and ECO-CH-AR.

#### Material examined

Mexico, Quintana Roo, Laguna Guerrero, 8 m asl [18.6920, -88.2615], coll. Pérez-Lachaud G., 29-July-2020, in a colony of *C. rectangularis* established in a trap-nest (Hymenoptera: Formicidae), 1-F, holotype, CNAC 012528; same locality, collector, collection date, and source, 1-M, OSAL 159512; 1-DN, OSAL 159513; 1-DN, OSAL 159514; 1-F; OSAL 159515; 1-F, OSAL 159516; 1-M, OSAL 159517; 1-F, OSAL 159518; 1-F, OSAL 159519; 1-F, ECO-CH-AR AA3477; 1-F, ECO-CH-AR AA3478; same locality, collector, 20-September-2020, *C. rectangularis*, colony in a bamboo trap-nest, 2-F, in 75% ethanol, OSAL 160583.

#### Etymology

The species epithet “*spatulatus*” was chosen on the basis of the shape of most dorsal and opisthogastral setae.

#### Notes on the species

*Myrmozercon spatulatus* sp. nov. also belongs in *Myrmozercon sensu lato* especially based on the long legs with hypertrichous setae and the reticulate dorsal shield. Within this species group, the absence of metasternal setae is similar to *M. iainkayi* Walter (Walter, 2003) and *M. beardae* Shaw & Seeman (Shaw & Seeman, 2009). *Myrmozercon iainkayi* can be differentiated by several characters, *e.g.*, the presence of a hypertrichous dorsal shield with simple and short setae (*M. spatulatus* sp. nov. has 27–31 pairs of moderately long and spatulate setae), coxae I–IV with 6, 6, 6, and 4 setae respectively (with standard set of coxal setae in *M. spatulatus* sp. nov.), and a horseshoe-shaped sternal shield (only slightly concave posteriorly in the new species). *Myrmozercon spatulatus* sp. nov. differs from *M. beardae* by the different shape of the sternal, epigynal, and anal shields, in addition to characters like the shape and number of setae on the dorsal shield (23–25 pairs of moderately short and simple setae in *M. beardae,* 27–31 pairs of moderately long and spatulate setae in *M. spatulatus* sp. nov.), and different setation on coxa IV (2 setae in *M. beardae*, 1 in *M. spatulatus* sp. nov.).

### Molecular analysis

Out of the five individuals extracted, only the two *M. spatulatus* sp. nov. specimens yielded CO1 fragment sequences with a length of 550 bp (BOLD: AFX7255). DNA obtained from *Myrmozercon serratus* sp. nov. did not meet barcode compliance standards.

**Table 3:** *Camponotus* spp. colony composition and occurrence of *Myrmozercon* mites at Laguna Guerrero, Quintana Roo, Mexico.

		**Ant host:** ***Camponotus atriceps***							**Mite:** ***Myrmozercon serratus*** **sp. nov.**				
**Collecting date**	**Nesting microhabitat**	**Queen**	**Gynes**	**Workers**	**Males**	**Larvae**	**Pupae**	**Colony size** ^*^	**Adults**	**Larvae**	**Protonymphs**	**Deutonymphs**	**Total**
17/08/2019	dead wood	0	10	57	0	0	0	67	8	1	0	3	12
14/05/2020	dry branch on the ground	0	3	30	0	9	23	56	0	0	0	1	1
10/08/2020	trap nest	0	2	82	0	2	0	84	7	0	0	3	10
01/08/2021	dry branch still on a *Hibiscus* shrub	0	9	87	14	19	0	110	12	4	3	12	31
15/08/2021	trap nest	0	8	165	23	2	6	202	24	2 (a)	4	19	49
22/08/2021	*M. tibicinis* dry pseudobulb	0	0	7	0	0	6	13	0	0	0	0	0
12/09/2021	*M. tibicinis* dry pseudobulb	0	5	111	2	0	0	118	12	0	0	9	21
15/01/2022	dry hanging branch	0	0	50	0	47	3	53	16 (b)	0	0	0	16
24/01/2022	trap nest	0	2	79	0	1	0	81	3	0	0	0	3
29/01/2022	trap nest	0	0	176	0	50	55	231	27	0	0	0	27
12/02/2022	*M. tibicinis* dry pseudobulb	0	0	208	0	0	0	208	9	1	0	0	10
26/06/2022	trap nest	0	12	165	38	310	70	285	16	2	2	2	22
17/07/2022	trap nest	1	9	72	8	0	0	90	17	3	0	2	22
**Total**		**1**	**60**	**1,289**	**85**	**440**	**163**	**1,598**	**151**	**13**	**9**	**51**	**224**
		**Ant host:** ***Camponotus rectangularis***							**Mite:** ***Myrmozercon spatulatus*** ** sp. nov.**				
**Collecting date**	**Nesting microhabitat**	**Queen**	**Gynes**	**Workers**	**Males**	**Larvae**	**Pupae**	**Colony size** ^*^	**Adults**	**Larvae**	**Protonymphs**	**Deutonymphs**	**Total**
29/03/2020	dry branch still on a tree	0	0	18	0	112	17	35	0	0	0	0	0
30/04/2020	*M. tibicinis* dry pseudobulb	1	0	246	0	367	137	384	0	0	0	0	0
14/05/2020	dry branch still on a tree	1	7	535	277	189	468	1,288	0	0	0	0	0
21/06/2020	dry hanging branch	0	16	35	2	11	15	68	1	0	0	0	1
11/07/2020	palma chit stem	0	10	13	0	1	0	23	0	0	0	1	1
29/07/2020	trap nest	1	53	105	6	29	33	198	16	3 (c)	1	6	26
10/08/2020	trap nest	1	2	55	11	11	3	72	4	1 (d)	0	0	5
20/09/2020	trap nest	0	0	57	19	14	11	87	6	0	0	0	6
02/07/2023	trap nest	0	0	36	0	103	52	88	0	0	0	0	0
**Total**		**4**	**88**	**1,100**	**315**	**837**	**736**	**2,243**	**27**	**4**	**1**	**7**	**39**

**Notes.**

**Figure 13:**
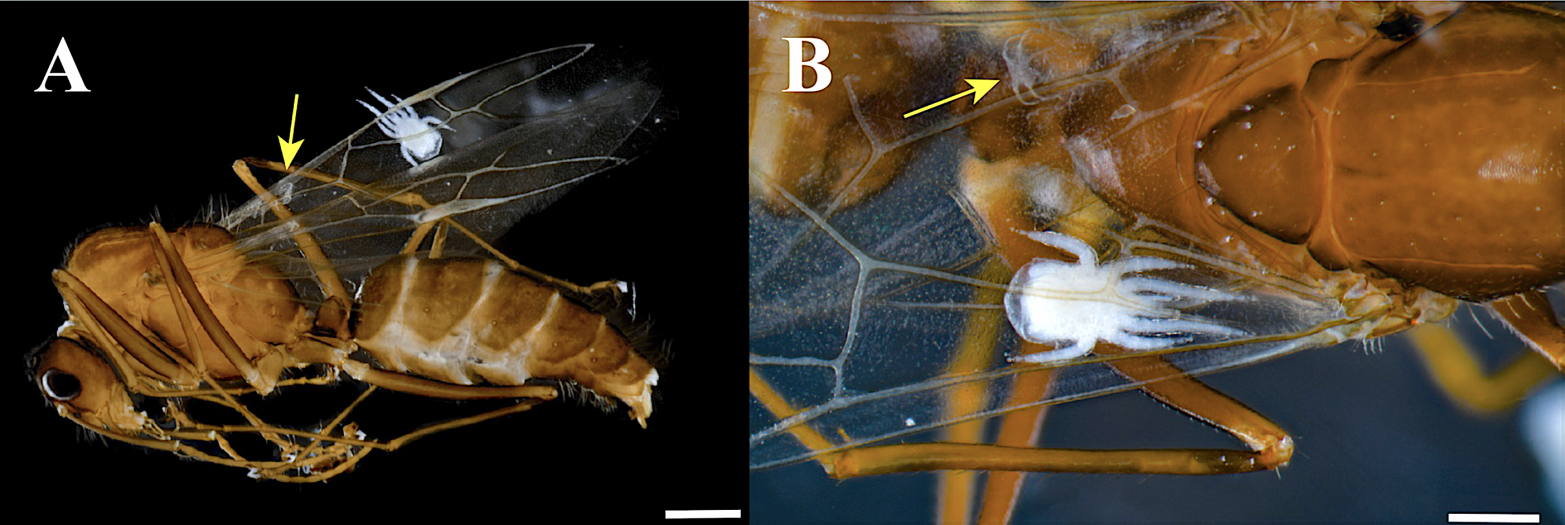
*Myrmozercon serratus* sp. nov., larva. (A) Larva on the wing of a *Camponotus atriceps* male. Note that the larva was dislodged from its initial, fixed position on the wing, between the veins. Scale bar: one mm. (B) Close-up, larva attached to the wings of a male ant, in original position. Scale bar: 0.5 mm. Arrows: possible remnants of exuviae. Photos credit: Humberto Bahena-Basave.

* Colony size without larvae. (a) one larva attached to a male wing (see Fig. 13); one larva recovered from the ethanol; (b) one adult mite under the head of a worker; (c) three larvae attached to the wings of three gynes; (d) one larva retrieved from the ethanol.

### Host associations and mite incidence

A total of 13 complete colonies or samples of *C. atriceps*, and nine of *C*. *rectangularis* were examined (Table 3). Additionally, samples of four other arboreal ant species collected in the same area were also examined (see below, Table S1). *Myrmozercon* mites were only found in the colonies and nests of the two *Camponotus* species. *Myrmozercon serratus* sp. nov. was only found associated with *C. atriceps* colonies, while *M. spatulatus* sp. nov. was exclusive to colonies of *C*. *rectangularis*. Most developmental instars of the mites were retrieved except for eggs. A total of 224 *M. serratus* mites (151 adults of both sexes, 13 larvae, nine protonymphs, and 51 deutonymphs) and 39 *M. spatulatus* (27 adults of both sexes, four larvae, one protonymph, and seven deutonymphs) were secured. We observed four cases where larvae were attached to the wings of sexuals: three cases in *C. rectangularis* and one case in *C. atriceps* (Table 3, Fig. 13). All other larvae were retrieved from the ethanol. Interestingly, in cases where larvae were detached from the wings, what might be exuviae or chorion traces remained stuck on the wings (Fig. 13, arrows). These observations seem to indicate that females lay eggs or give birth to larvae on winged individuals.

*Camponotus atriceps* was the most frequently encountered ant species at the study site; it also showed the highest mite infestation (Table 3), with mites present in 12 out of 13 samples (92%) and up to 49 mites in the most infested colony. In contrast, mites were present in only five *C. rectangularis* samples (56%, *n* = 9), only one of them being heavily infested. The choice of ant nesting site, artificial trap-nests or nests inside dead branches or dried orchid pseudobulbs, did not seem to affect the likelihood of ant-mite associations. In contrast, the abundance of winged sexuals was significantly correlated with mite abundance in the case of *C. atriceps*, the only species for which sample size allowed a test of this hypothesis (Fig. S6, *R*^2^ = 0.355 *F*_1,11_ = 15.8, *p* < 0.05). Colonies of other arboreal ant species (*Crematogaster crinosa* Mayr, *Pseudomyrmex gracilis* (Fabricius), *Dolichoderus lutosus* (F. Smith), and *Cephalotes porrasi* (Wheeler)) from the same locality, harbored no *Myrmozercon* mites, but some unidentified mesostigmatid mites were found in association with *C. crinosa* and *D. lutosus* (Table S1).

**Table 4:** Ant hosts of *Myrmozercon* spp. mites.

**Species**	**Referred to as**	**Ant host**	**Host subfamily**	**Host tribe**	**Locality**	**References**
*Myrmozercon aequalis* (Banks)	*Myrmonyssus aequalis*	*Iridomyrmex gracilis* (Lowne)	Dolichoderinae	Leptomyrmecini	Tasmania and Australia	Banks (1916)
*Myrmozercon andongensis* Joharchi, Jung & Keum		*Camponotus japonicus* Mayr	Formicinae	Camponotini	Korea	Joharchi, Jung & Keum (2018)
*Myrmozercon antennophoroides* (Berlese)	*Myrmonyssus antennophoroides*	*Camponotus aethiops* (Latreille)	Formicinae	Camponotini	Italy	Berlese (1904)
*Myrmozercon beardae* Shaw & Seeman		Unidentified ants	??	??	Southern Australia	Shaw & Seeman (2009)
*Myrmozercon brachiatus* (Berlese)	*Myrmonyssus brachiatus*	*Messor minor* (André) *Messor mediosanguineus* Donisthorpe	Myrmicinae	Stenammini	Italy Iran	Berlese (1903) Khalili-Moghadam & Babaeian (2023)
*Myrmozercon brachytrichos* Joharchi, Arjomandi & Trach		*Crematogaster inermis* Mayr	Myrmicinae	Crematogastrini	Iran	Joharchi, Arjomandi & Trach (2017)
*Myrmozercon brevipes* (Berlese)	*Myrmozercon ovatum*	*Tapinoma nigerrimum* (Nylander), ^*^Tapinoma erraticum (Latreille) *Cataglyphis emeryi* (Karawajew)	Dolichoderinae Formicinae	Tapinomini Formicini	Italy Greece Turkmenistan Iran	Berlese (1902), Berlese (1904) Kontschán & Seeman (2015) Ghafarian et al. (2013) Karawajew (1909)
*Myrmozercon burwelli* Shaw & Seeman		*Polyrhachis flavibasis* Clark	Formicinae	Camponotini	Eastern Australia	Shaw & Seeman (2009)
*Myrmozercon chapmani* (Baker & Strandtmann)	*Myrmonyssus chapmani*	Host unknown (intercepted in an orchid)	–	–	from Mexico? (intercepted at Laredo, USA)	Baker & Strandtmann (1948)
*Myrmozercon clarus* (Hunter & Hunter)	*Myrmonyssus clarus*	*Crematogaster laeviuscula* Mayr	Myrmicinae	Crematogastrini	Georgia, USA	Hunter & Hunter (1963)
*Myrmozercon crinitus* Joharchi		*Pheidole pallidula* (Nylander)	Myrmicinae	Attini	Iran	Joharchi & Moradi (2013)
*Myrmozercon cyrusi* Ghafarian & Joharchi		*Monomorium* sp.	Myrmicinae	Solenopsidini	Iran	Ghafarian et al. (2013)
*Myrmozercon diplogenius* (Berlese)	*Myrmonyssus diplogenius*	*Camponotus aethiops*	Formicinae	Camponotini	Italy	Berlese (1903), Berlese (1904)
*Myrmozercon eidmanni* (Sellnick)	*Myrmonyssus eidmanni*	*Crematogaster impressa* Emery	Myrmicinae	Crematogastrini	Bioko Island, Equatorial Guinea	Sellnick (1941)
*Myrmozercon hunteri* Joharchi, Babaeian & Seeman		*Myrmica* sp.	Myrmicinae	Myrmicini	Iran	Joharchi, Babaeian & Seeman (2015)
*Myrmozercon iainkayi* Walter		*Polyrhachis* sp.; *Polyrhachis australis* Mayr	Formicinae	Camponotini	Eastern Australia	Walter (2003) and Shaw & Seeman (2009)
*Myrmozercon karajensis* Joharchi, Halliday, Saboori & Kamali		*Camponotus* sp.	Formicinae	Camponotini	Iran	Joharchi et al. (2011)
*Myrmozercon kishidai* Joharchi, Saito, Muto & Kinomura		*Crematogaster teranishii* Santschi	Myrmicinae	Crematogastrini	Japan	Joharchi et al. (2023)
*Myrmozercon liguricus* (Vitzthum)	*Myrmonyssus liguricus*	*Crematogaster scutellaris* (Olivier)	Myrmicinae	Crematogastrini	Italy	Vitzthum (1930)
*Myrmozercon minor* (Sellnick)	*Myrmonyssus minor*	*Crematogaster impressa*	Myrmicinae	Crematogastrini	Bioko Island, Equatorial Guinea	Sellnick (1941)
*Myrmozercon patagonicus* Trach & Khaustov		*Camponotus* sp.	Formicinae	Camponotini	Patagonia and Chile	Trach & Khaustov (2018)
*Myrmozercon rotundiscutum* Rosario & Hunter		*Camponotus* sp.	Formicinae	Camponotini	Idaho, USA	Rosario & Hunter (1988)
*Myrmozercon scutellatus* (Hull)	*Myrmonyssus scutellatus*	*Iridomyrmex innocens* Forel	Dolichoderinae	Leptomyrmecini	Western Australia	Hull (1923)
*Myrmozercon spinosus* (Hunter & Hunter)	*Myrmonyssus spinosus*	*Crematogaster* sp.	Myrmicinae	Crematogastrini	Kansas, USA	Hunter & Hunter (1963)
*Myrmozercon sternalis* Babaeian, Joharchi & Saboori		*Formica* sp. *Tapinoma* sp.	Formicinae Dolichoderinae	Formicini Tapinomini	Iran	Babaeian, Joharchi & Saboori (2013) Khalili-Moghadam & Saboori (2015)
*Myrmozercon tauricus* Trach & Khaustov		*Crematogaster schmidti* (Mayr)	Myrmicinae	Crematogastrini	Ukraine	Trach & Khaustov (2011)
*Myrmozercon titan* (Berlese)	*Myrmonyssus titan*	Host unknown (reported as ‘surely myrmecophilous’)	–	–	East Africa	Berlese (1916)
*Myrmozercon yemeni* (Ueckermann & Loots)	*Parabisternalis yemeni*	Host unknown (from Malaise trap)	–	–	Yemen	Ueckermann & Loots (1995)
*Myrmozercon* sp.		†*Ctenobethylus goepperti* (Mayr)	Dolichoderinae	Tapinomini	Baltic amber	Dunlop et al. (2014)
*Myrmozercon serratus* sp. nov.		*Camponotus atriceps*	Formicinae	Camponotini	Quintana Roo and Chiapas, Mexico	This study
*Myrmozercon spatulatus* sp. nov.		*Camponotus rectangularis*	Formicinae	Camponotini	Quintana Roo, Mexico	This study

**Notes.**

* The mention of the host *Tapinoma erraticum* (Latreille) is probably a misidentification for *T. nigerrimum* according to Kontschán & Seeman (2015).

## Discussion

### Host specificity

Most *Myrmozercon* species are known to be associated with, or have been collected with ants (Table 4) (Shaw & Seeman, 2009; Trach & Khaustov, 2011; Joharchi & Moradi, 2013; Kontschán & Seeman, 2015; Joharchi, Babaeian & Seeman, 2015;  Joharchi, Arjomandi & Trach, 2017; Joharchi, Jung & Keum, 2018; Joharchi et al., 2023). Even the undescribed *Myrmozercon* sp. fossil was found in the same inclusion as its host (†*Ctenobethylus goepperti*) (Dunlop et al., 2014). The observation that each of the two new species appears to be exclusively associated with their own ant host species fits well with existing data on host associations in the genus *Myrmozercon*. Detailed hosts association records are often absent in Laelapidae, but in *Myrmozercon*, the ant host species is now known for 27 of the 31 known species (not for *M*. *beardae*, *M*. *chapmani*, *M*. *titan* (Berlese), and *M. yemeni* (Ueckermann & Loots)) (Table 4). These *Myrmozercon* ant hosts belong to 12 genera (11 extant, 1 extinct) in three subfamilies: Formicinae (*Camponotus*, *Cataglyphis*, *Polyrhachis*, *Formica*), Dolichoderine (*Iridomyrmex*, *Tapinoma*, †*Ctenobethylus*), and Myrmicinae (*Crematogaster*, *Messor*, *Monomorium*, *Myrmica*, *Pheidole*) (Table S2). The members of two ant genera with arboreal nesting habits seem particularly susceptible to *Myrmozercon* mites: including records in this study, seven species of *Camponotus* (Formicinae: Camponotini) and seven of *Crematogaster* (Myrmicinae: Crematogastrini) are known hosts for mites in this genus (Table S2) suggesting some ecological component to the associations.

Most *Myrmozercon* species are known from a single host species, including our two new records (21 two-way relationships). There are only two cases where a single ant host species is associated with two different, sympatric *Myrmozercon* species: *Camponotus aethiops* (Latreille) hosts *M. antennophoroides* and *M. diplogenius* (Berlese) in Italy, and *Crematogaster impressa* Emery hosts *M*. *eidmanni* (Sellnick) and *M*. *minor* (Sellnick) in Bioko Island (Table 4). Conversely, in a few cases, a *Myrmozercon* species may be associated with two species of the same ant genus, such as *M. brachiatus* (Berlese) with *Messor minor* (André) and *Messor mediosanguineus* Donisthorpe, or with two species of different genera that furthermore belong to two different subfamilies (Formicinae and Dolichoderinae), such as *M. brevipes* (Berlese) with *Tapinoma nigerrimum* (Nylander) and *Cataglyphis emeryi* (Karawajew) and *M. sternalis* Babaeian, Joharchi & Saboori with *Formica* sp. and *Tapinoma* sp. However, it should be noted that these associations with two different ant species occur in different regions of a same country or in different countries. The current material was recovered from congeneric hosts, collected in the same locality, in the same time frame, with both hosts using similar nesting microhabitats. This strongly suggests one-on-one host specificity. Available data is inconsistent with the hypothesis that *Myrmozercon* is locally host specific but variable in its host choice across its geographic range, given the observation that the same species of *Myrmozercon* found on *C. atriceps* in Quintana Roo (Caribbean coast) was also recovered from *C. atriceps* from Chiapas, on the other side of Mexico (Pacific coast).

As a side note, mites associated with a third *Camponotus* species (*C. planatus* Roger), were collected in southwestern Quintana Roo, but at a different locality. These specimens belong to yet another new species of *Myrmozercon*. Description was not attempted because of the small number of available specimens and the fact that they were from a different locality. Overall, current results confirm, and even reinforce, a general pattern of host specificity for members of the genus. They also suggest the likelihood of a substantially higher diversity of species of *Myrmozercon* than previously suspected.

### Biogeography

Although of cosmopolitan distribution, the New World *Myrmozercon* fauna has rarely been the focus of diversity studies. Four *Myrmozercon* species have been described from the Neartic region, the previously mentioned *M. chapmani* from Mexico (no host association, uncertain locality), *M. clarus* Hunter & Hunter from Athens, Georgia, U.S.A., *M*. *spinosus* Hunter & Hunter from Kansas, U.S.A. (Hunter & Hunter, 1963), and *M*. *rotundiscutum* Rosario & Hunter from Idaho, U.S.A. (Rosario & Hunter, 1988). A species described from Patagonia was recorded as the first record of this genus from the Neotropics (Trach & Khaustov, 2018), but strictly speaking our records are the first for this biogeographic region given that Patagonia corresponds to the Andean zone, not to the Neotropics *s.s*. (Morrone, 2015). According to Noguera-Urbano & Escalante (2017) and Escalante & Morrone (2020), Neotropics *s.s.* extends only from the lowland areas of Veracruz and the Pacific coasts of Mexico to the southern edge of Amazonia in Brazil, between 20°N–15°S; *M. patagonicus* was collected much farther south than these limits (51°56′55.2″S, 72°23′24.8″W; Trach & Khaustov, 2018).

### Female egg laying and larval behavior

Four larvae of *Myrmozercon* (one of *M. serratus* and three of *M. spatulatus*) were observed on the wings of alates (a male for *M. serratus*, three gynes for *M. spatulatus*). The larvae were under the proximal part of the forewing, between the wing veins, with legs I and II directed forward. Their body shape and position match the space between the wing veins (Fig. 13B). These observations could be accidental, but the presence of four out of 17 recovered larvae on the wings of alates is unusual. Moreover, the remaining larvae were recovered from alcohol filled vials of colonies that nearly always included alates, so an association with this caste for additional larvae cannot be excluded. These are the first records of *Myrmozercon* larvae on the wings of alates. Reasons why this phenomenon has not been reported before could be either because the behavior is limited to the newly described species of Neotropical *Myrmozercon*, or because previous studies did not include close examination of complete ant colonies and/or of alate wings.

Assuming the presence of larvae on the wings is not accidental, this raises questions which cannot be answered definitively, but which may be worth asking to stimulate future research. How do these larvae end up on the wing, and second, is there a functional importance to this? Concerning the first question, the larvae are poorly developed, and it seems unlikely that they would be able to crawl on alates themselves. Most likely, females deposit eggs containing fully grown larvae on the wing of the ants, a hypothesis supported by the presence of what looks like chorion remnants on the wings (Fig. 13). Accidental deposition of larvae on the wing, *e.g.*, phoretic females giving birth on the wing due to the shock of being deposited in alcohol, has to be rejected because it cannot explain why some larvae were found in a very specific position at the base of the wing. The assumption of ovovivipary in *Myrmozercon* is based on a suggestion to that effect by Sellnick (1941). Notably, we are unaware of any data confirming whether *Myrmozercon* is oviparous or ovoviviparous (or both; at least one other laelapid, *Hypoaspis larvicolus* Joharchi & Halliday can be both (Cakmak & Da Silva, 2018)). Which leaves the second question, is the presence of larvae on the wings of alates of functional importance? We consider two options, a role in passive co-dispersal with the host or a refuge from predation. Dispersal would seem a logical possibility but dispersal in most Laelapidae, including ant associates (*e.g.*, Walter & Moser, 2010), involves mainly adults (deutonymphs may be involved in some other gamasine families). The same has been observed in *Myrmozercon* species, *e.g.*, *Myrmozercon iainkayi* adults have been collected on both workers and alates of *Polyrachis* sp. in Queensland, Australia (Walter, 2003) and adults of *M. liguricus* (Vitzthum) were recovered from winged sexuals of *Crematogaster scutellaris* (Olivier) in Italy (Vitzthum, 1930). In fact, adults have been reported as clinging to the host ants in seven *Myrmozercon* species (most likely an undercount because such behavior can easily go unnoticed in populous host societies). On the other hand, dispersal of ant associates is not restricted to dispersal with new founding queens, and most dispersal may involve movement of several or even all developmental stages (adult and immatures) with their hosts when ants relocate their nest or move to satellite nests in polydomous species. In polydomous species the members of a single colony occupy at least two physically separated, but socially connected, nests. Several species of *Camponotus* and *Crematogaster*, including both arboreal and ground nesting species and the hosts of the newly described *Myrmozercon* species, are polydomous (Davidson et al., 2006; Martins Segundo et al., 2017; Soares Jr & Oliveira, 2021; Pérez-Lachaud & Lachaud, 2021).

The alternative “refuge” hypothesis is based on the observation that laelapid larvae are quite vulnerable. As noted above, larvae in Laelapidae are regressed, non-feeding, and short-lived (from 0.5–2 days in most species (Abou-Awad et al., 1989; Yoder, 1996; Cakmak & Da Silva, 2018)). In *Gromphadorholaelaps schaeferi* Till, an associate of the Madagascar hissing roach *Gromphadorhina portentosa* (Schaum), females give birth to larvae on the abdomen of the host to avoid interactions with other females who will attack and kill the larvae. After the larvae molt to protonymphs they seem to be big enough to return to the mite colony near the head of the roach (Yoder, 1996; Yoder, 1997). Could the situation in the Mexican *Myrmozercon* be similar in that larvae are deposited on the wings of alates to protect them until they molt to protonymphs? This hypothesis does make a number of assumptions. First, we assume there is a significant threat to the mite larvae, either from conspecific females, other myrmecophiles in the nest (*e.g.*, females of *Holostaspis* sp. (Laelapidae) were recovered from a nest containing *M. serratus*), or even worker ants, and second, as is the case with most ant species, that alates stay in the nest for extended periods; moreover, it is unclear if the larvae could hold on a flying ant. On the other hand, unlike the dispersal hypothesis, which requires an entirely new function for larval Laelapidae, this hypothesis is consistent with what we know about this regressed instar.

Deciding among the various explanations for the presence of larvae on the ant alates, will require additional detailed observations and/or experimental work.

## Supplemental Information

10.7717/peerj.18197/supp-1 Supplemental Information 1 Study site vegetation

10.7717/peerj.18197/supp-2 Supplemental Information 2 Mangrove trees at the border of the lagoon Photo credit: Gabriela Pérez-Lachaud.

10.7717/peerj.18197/supp-3 Supplemental Information 3 Example of vegetation on the study site Indigenous trees and palms (A–B) intermixed with coconut palm trees (C). Photos credit: Jean-Paul Lachaud.

10.7717/peerj.18197/supp-4 Supplemental Information 4 *Myrmozercon serratus* sp. nov. Female, dorsal view Photo credit: Manuel Elías-Gutiérrez & Gabriela Pérez-Lachaud.

10.7717/peerj.18197/supp-5 Supplemental Information 5 *Myrmozercon serratus* sp. nov. Female, anal plate Photocredit: Manuel Elías-Gutiérrez & Gabriela Pérez-Lachaud.

10.7717/peerj.18197/supp-6 Supplemental Information 6 *Myrmozercon serratus* sp. nov. Female, gnathosoma Photo credit: Manuel Elías-Gutiérrez & Gabriela Pérez-Lachaud.

10.7717/peerj.18197/supp-7 Supplemental Information 7 Correlation between the number of males and gynes in colonies of *Camponotus atriceps* and abundance of *Myrmozercon serratus* sp. nov. mites (n = 13) Photo credit: Humberto Bahena-Basave.

10.7717/peerj.18197/supp-8 Supplemental Information 8 Additional arboreal ant species surveyed at Laguna Guerrero, Quintana Roo, Mexico *Colony size without larvae.

10.7717/peerj.18197/supp-9 Supplemental Information 9 Summary of *Myrmozercon* spp. - ant host associations *Same *Myrmozercon* species involved.

## Additional Information and Declarations

### Field Study Permissions

The following information was supplied relating to field study approvals (i.e., approving body and any reference numbers): Collecting and field work comply with the current laws of Mexico. The Secretaria de Medio Ambiente y Recursos Naturales (SEMARNAT, Secretary of Environment and Natural Resources of Mexico), approved field sampling under the collection permit FAUT-0277 granted to GP-L.

### DNA Deposition

The following information was supplied regarding the deposition of DNA sequences: The two species described here are available at BOLDSYSTEMS: BOLD:AFX7255.
https://bench.boldsystems.org/index.php/Public_BarcodeCluster?clusteruri=BOLD:AFX7255.

### Data Availability

The following information was supplied regarding data availability: The complete data on parasitism by the two myrmecophilous mite species studied are available in Table 3.

### New Species Registration

The following information was supplied regarding the registration of a newly described species: Publication LSID: urn:lsid:zoobank.org:pub:93B2E7CB-3C56-4C21-922A-B4B6FCABB3DF *Myrmozercon serratus*: urn:lsid:zoobank.org:act:AB2A11A9-C21D-4C0F-99A7-36FD21854D8D *Myrmozercon spatulatus*: urn:lsid:zoobank.org:act:50BA702F-CCB1-488D-A3AC-0618B9F5881A.
