# Supplementary material for: Myrmozercon mites are highly host specific: two new species of Myrmozercon Berlese associated with sympatric Camponotus ants in southern Quintana Roo, Mexico
Source: PeerJ. 2024 Oct 25;12:e18197. doi: 10.7717/peerj.18197 (PMC11514769; doi:10.7717/peerj.18197)
Supplement: Supplemental Information 1 [file peerj-12-18197-s001.docx]

**Supplementary Material S1**

**Study site vegetation**

The vegetation on the study site, in southern Quintana Roo, 30 km north of Belize, consists mainly of mangrove trees [*Rhizophora mangle* Linnaeus (Rhizophoraceae), *Conocarpus erectus* Linnaeus, and *Laguncularia* *racemosa* (Linnaeus) C.F. von Gärtner (Combretaceae)] which border the lagoon (Fig. S1). Indigenous trees and palms [*Coccoloba* *uvifera* (Linnaeus) Linnaeus (Polygonaceae), *Guazuma ulmifolia* Lamarck (Malvaceae), *Lysiloma latisiliquum* (Linnaeus) Bentham (Fabaceae), *Manilkara zapota* (Linnaeus) P. Royen (Sapotaceae), *Piscidia piscipula* (Linnaeus) Sargent (Fabaceae), *Bucida buceras* Linnaeus (Combretaceae), *Metopium* *brownei* (von Jacquin) Urban (Anacardiaceae), and *Thrinax radiata* Loddiges ex Schultes & Schultes (Arecaceae)] (see Fig. S2a, b) are intermixed with coconut palm trees [*Cocos* *nucifera* Linnaeus (Arecaceae)] (Fig. S2c) and ornamental plants [e.g., black bamboo *Phyllostachys nigra* (Loddiges ex Lindley) Munro (Poaceae), *Bougainvillea* spp. (Nyctaginaceae) and *Hibiscus syriacus* Linnaeus (Malvaceae)].
