# Supplementary figures and images for: Myrmozercon mites are highly host specific: two new species of Myrmozercon Berlese associated with sympatric Camponotus ants in southern Quintana Roo, Mexico
Source: PeerJ. 2024 Oct 25;12:e18197. doi: 10.7717/peerj.18197 (PMC11514769; doi:10.7717/peerj.18197)

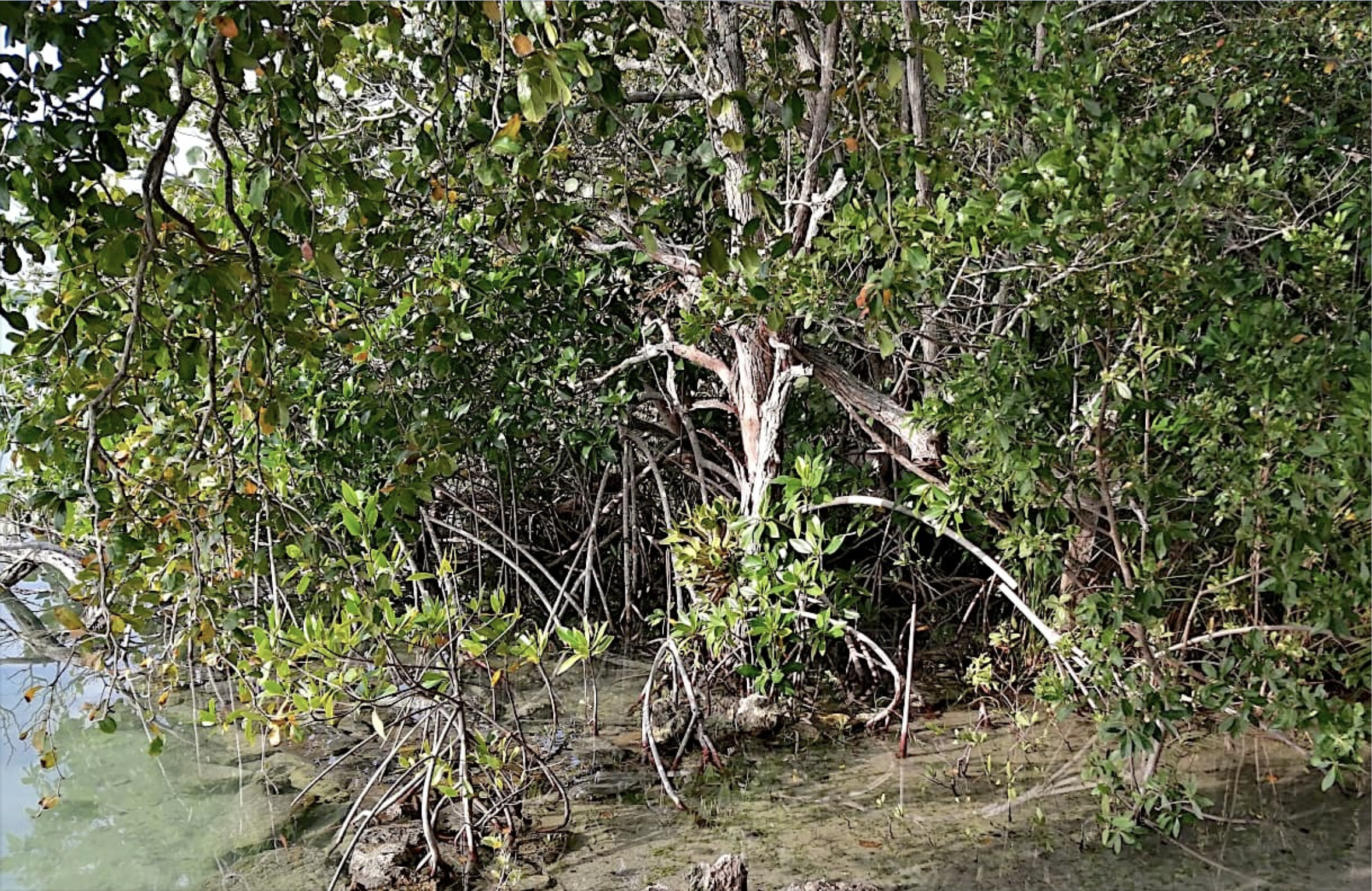

Supplement: Supplemental Information 2 — Photo credit: Gabriela Pérez-Lachaud. [file peerj-12-18197-s002.png]

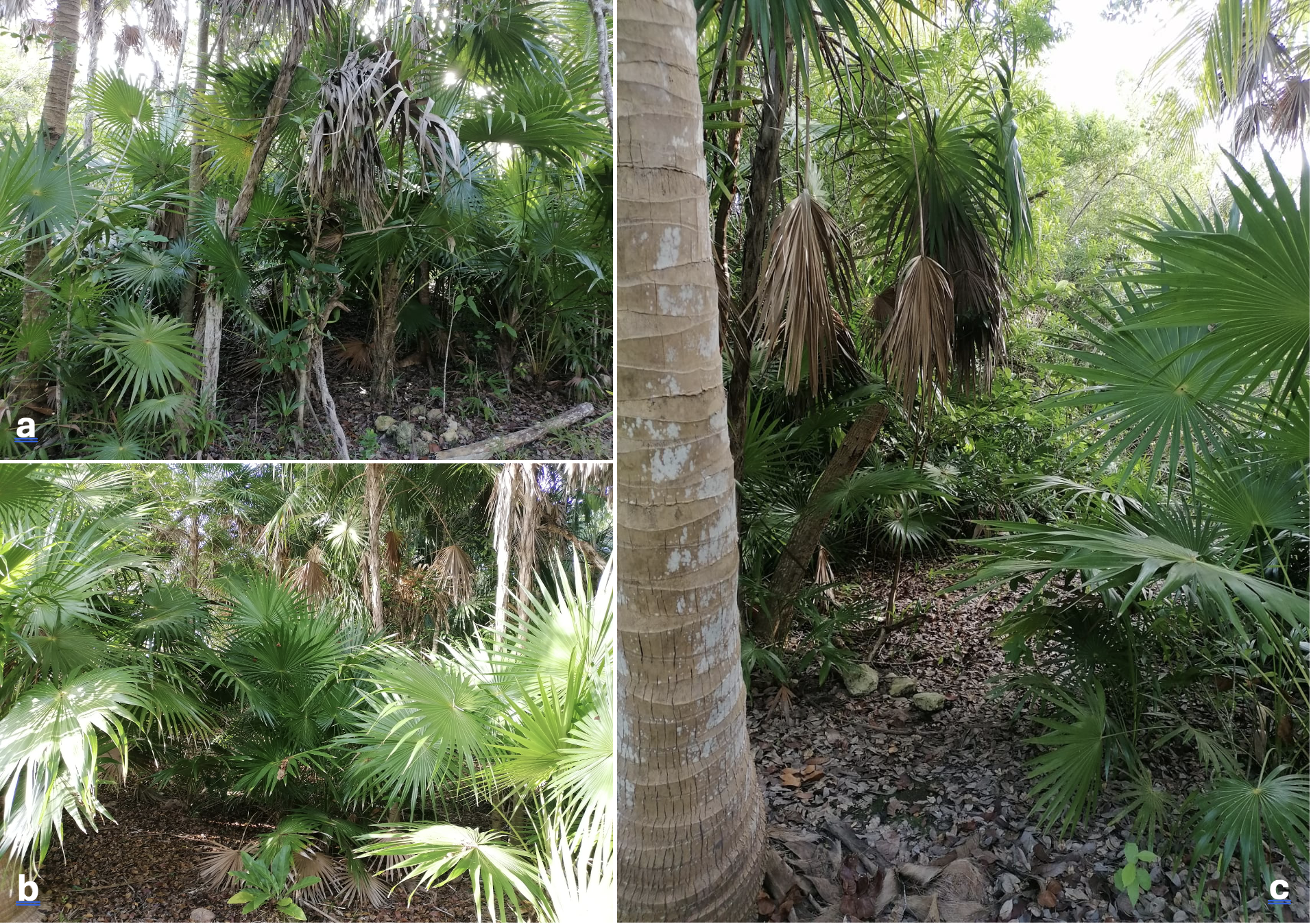

Supplement: Supplemental Information 3 — Indigenous trees and palms (A–B) intermixed with coconut palm trees (C). Photos credit: Jean-Paul Lachaud. [file peerj-12-18197-s003.png]

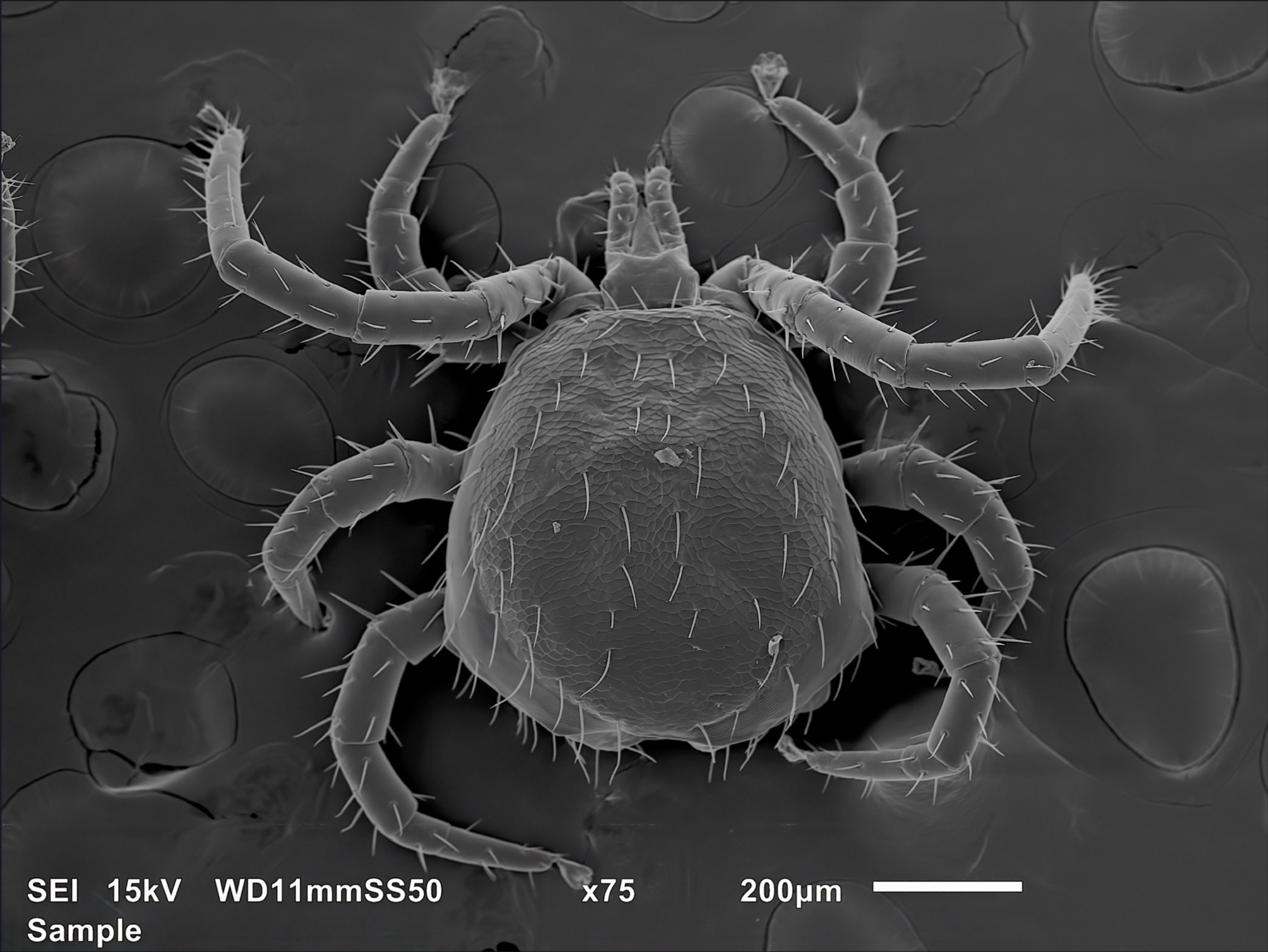

Supplement: Supplemental Information 4 — Photo credit: Manuel Elías-Gutiérrez & Gabriela Pérez-Lachaud. [file peerj-12-18197-s004.png]

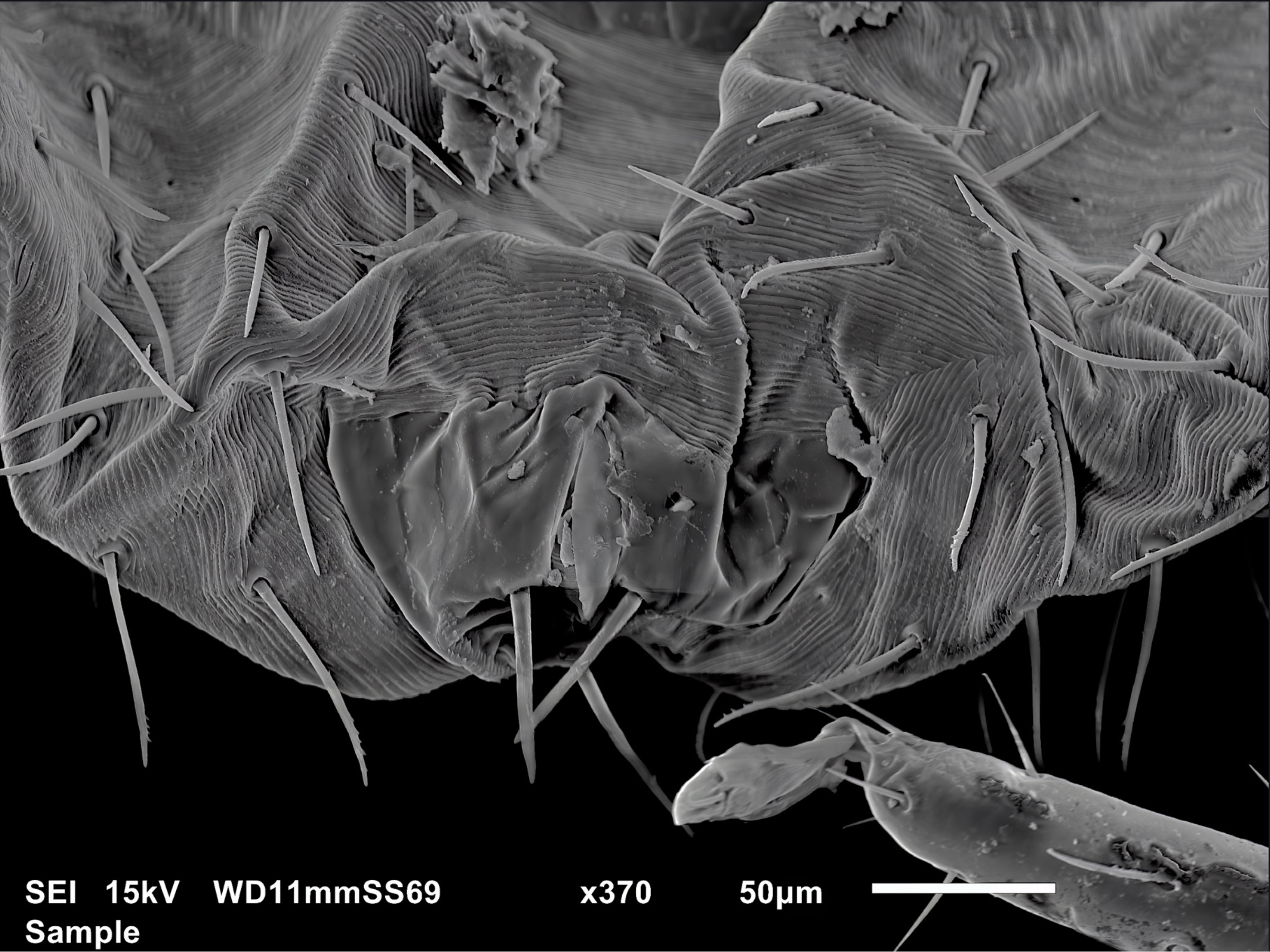

Supplement: Supplemental Information 5 — Photocredit: Manuel Elías-Gutiérrez & Gabriela Pérez-Lachaud. [file peerj-12-18197-s005.png]

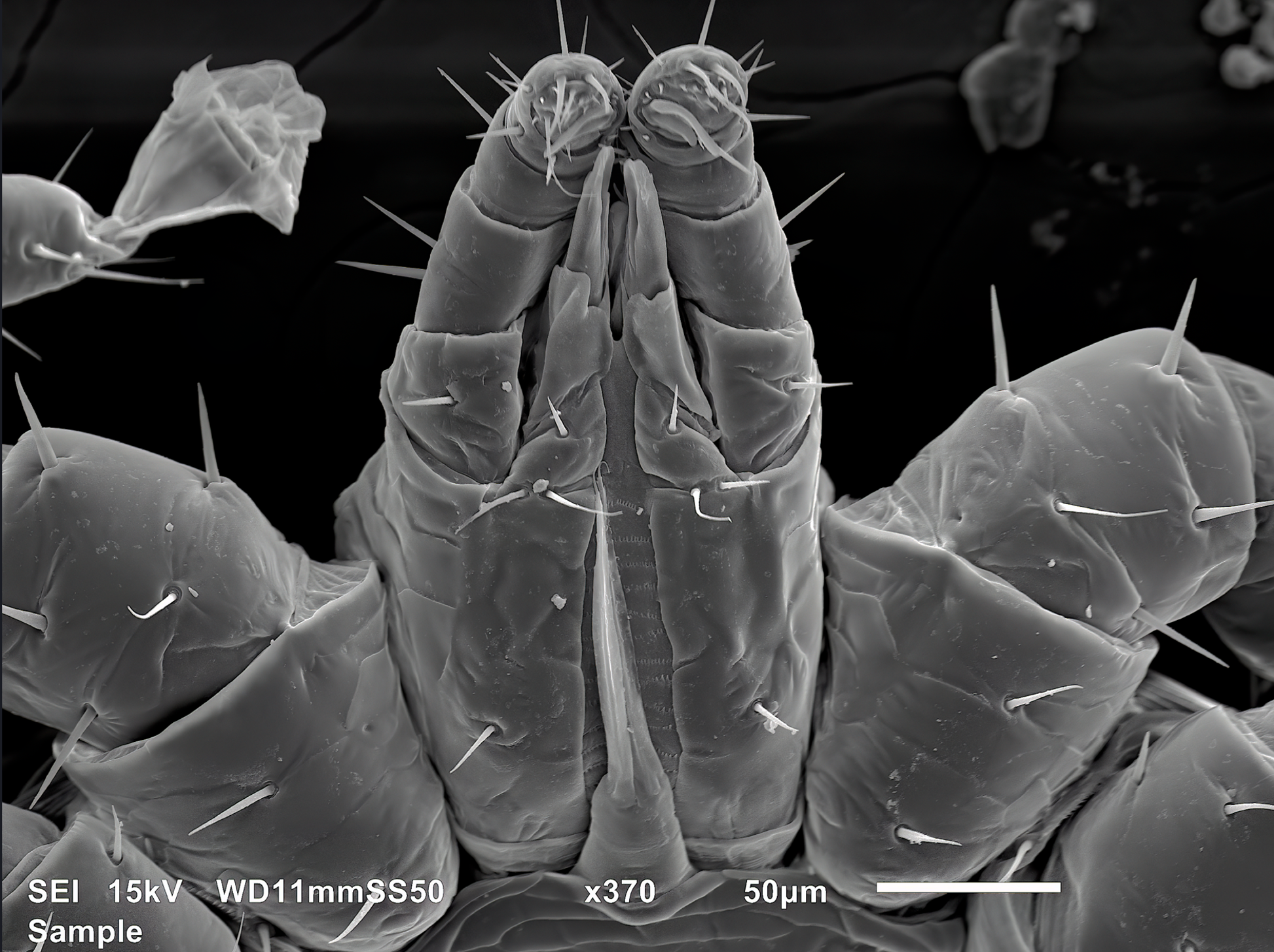

Supplement: Supplemental Information 6 — Photo credit: Manuel Elías-Gutiérrez & Gabriela Pérez-Lachaud. [file peerj-12-18197-s006.png]

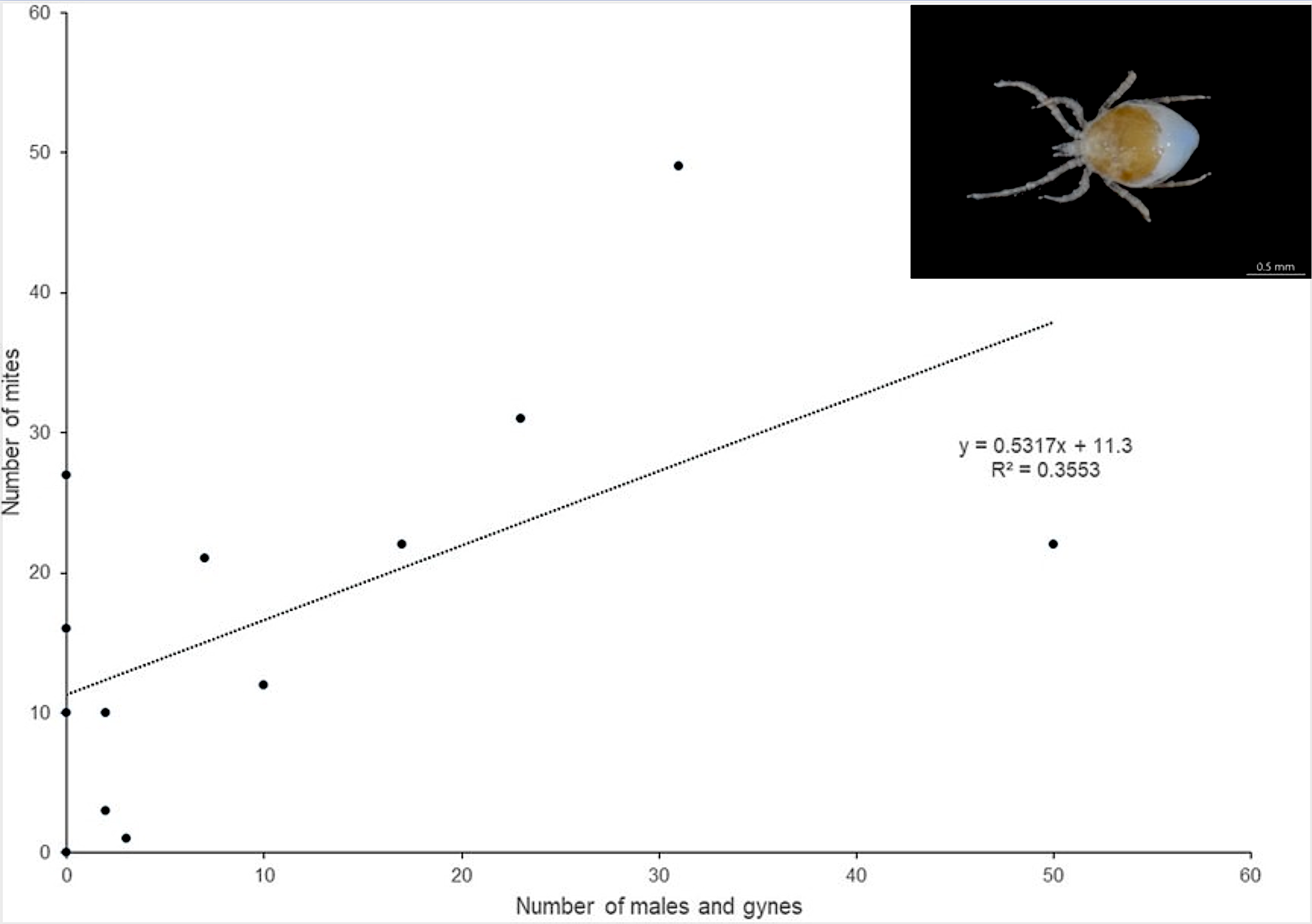

Supplement: Supplemental Information 7 — Photo credit: Humberto Bahena-Basave. [file peerj-12-18197-s007.png]
