# Supplementary material for: Myrmozercon mites are highly host specific: two new species of Myrmozercon Berlese associated with sympatric Camponotus ants in southern Quintana Roo, Mexico
Source: PeerJ. 2024 Oct 25;12:e18197. doi: 10.7717/peerj.18197 (PMC11514769; doi:10.7717/peerj.18197)
Supplement: Supplemental Information 8 — *Colony size without larvae. [file peerj-12-18197-s008.docx]

| **Table S1 Additional arboreal ant species surveyed at Laguna Guerrero, Quintana Roo, Mexico.** | | | | | | | | | | |
| --- | --- | --- | --- | --- | --- | --- | --- | --- | --- | --- |
| **Ant species** | **Collecting date** | **Nesting micohabitat** | **Queen** | **Gynes** | **Workers** | **Males** | **Larvae** | **Pupae** | **Colony size*** | **Acari presence** |
| *Cephalotes porrasi* (Wheeler) | 26/03/2023 | dry branch | 1 | 9 | 50 | 0 | 26 | 0 | 60 | none |
| *Crematogaster crinosa* Mayr | 27/08/2022 | trap-nest | 0 | 0 | 152 | 0 | 0 | 18 | 152 | several Gamasina mites |
| *Dolichoderus lutosus* (F. Smith) | 05/07/2020 | aerial soil on palm | 0 | 4 | 326 | 14 | 93 | 121 | 465 | 1 Ixodidae |
| *D. lutosus* | 09/02/2024 | trap-nest | 1 | 0 | 454 | 0 | 328 | 111 | 566 | 2 small unidentified mites |
| *Pseudomyrmex gracilis* (Fabricius) | 27/06/2020 | dry branch | 1 | 0 | 43 | 0 | 164 | 30 | 74 | none |
| *P. gracilis* | 14/03/2021 | dry branch | 1 | 0 | 33 | 0 | 285 | 71 | 105 | none |

**Notes:**

*Colony size without larvae.
