# Supplementary material for: Myrmozercon mites are highly host specific: two new species of Myrmozercon Berlese associated with sympatric Camponotus ants in southern Quintana Roo, Mexico
Source: PeerJ. 2024 Oct 25;12:e18197. doi: 10.7717/peerj.18197 (PMC11514769; doi:10.7717/peerj.18197)
Supplement: Supplemental Information 9 — *Same Myrmozercon species involved. [file peerj-12-18197-s009.docx]

| **Table S2 Summary of *Myrmozercon* spp. - ant host associations.** | | | | |
| --- | --- | --- | --- | --- |
| **Subfamily** | **Tribe** | **Ant host genus** | **Number of ant host species** | **Number of mite species involved** |
| Formicinae | Camponotini | *Camponotus* | 7 | 8 |
|  |  | *Polyrhachis* | 2 | 2 |
|  | Formicini | *Formica* | 1 | 1 |
|  |  | *Cataglyphis* | 1 | 1* |
| Myrmicinae | Crematogastrini | *Crematogaster* | 7 | 8 |
|  | Stenammini | *Messor* | 2 | 1 |
|  | Solenopsidini | *Monomorium* | 1 | 1 |
|  | Myrmicini | *Myrmica* | 1 | 1 |
|  | Pheidolini | *Pheidole* | 1 | 1 |
| Dolichoderinae | Leptomyrmecini | *Iridomyrmex* | 2 | 2 |
|  | Tapinomini | *Tapinoma* | 1 | 1* |
|  |  | †*Ctenobethylus* | 1 | 1 |
| ? | ? | Unknown host(s) | - | 4 |

**Notes:**

*Same *Myrmozercon* species involved.
